# Navigating drug repurposing for Chagas disease: advances, challenges, and opportunities

**DOI:** 10.3389/fphar.2023.1233253

**Published:** 2023-07-27

**Authors:** Exequiel O. J. Porta, Karunakaran Kalesh, Patrick G. Steel

**Affiliations:** ^1^ Department of Chemistry, Durham University, Durham, United Kingdom; ^2^ School of Health and Life Sciences, Teesside University, Middlesbrough, United Kingdom; ^3^ National Horizons Centre, Darlington, United Kingdom

**Keywords:** Chagas disease, combination therapy, drug discovery, drug repositioning, drug repurposing, neglected tropical diseases, parasitic disease, *Trypanosoma cruzi*

## Abstract

Chagas disease is a vector-borne illness caused by the protozoan parasite *Trypanosoma cruzi* (*T. cruzi*). It poses a significant public health burden, particularly in the poorest regions of Latin America. Currently, there is no available vaccine, and chemotherapy has been the traditional treatment for Chagas disease. However, the treatment options are limited to just two outdated medicines, nifurtimox and benznidazole, which have serious side effects and low efficacy, especially during the chronic phase of the disease. Collectively, this has led the World Health Organization to classify it as a neglected disease. To address this problem, new drug regimens are urgently needed. Drug repurposing, which involves the use of existing drugs already approved for the treatment of other diseases, represents an increasingly important option. This approach offers potential cost reduction in new drug discovery processes and can address pharmaceutical bottlenecks in the development of drugs for Chagas disease. In this review, we discuss the state-of-the-art of drug repurposing approaches, including combination therapy with existing drugs, to overcome the formidable challenges associated with treating Chagas disease. Organized by original therapeutic area, we describe significant recent advances, as well as the challenges in this field. In particular, we identify candidates that exhibit potential for heightened efficacy and reduced toxicity profiles with the ultimate objective of accelerating the development of new, safe, and effective treatments for Chagas disease.

## 1 Introduction

**FIGURE 1:**
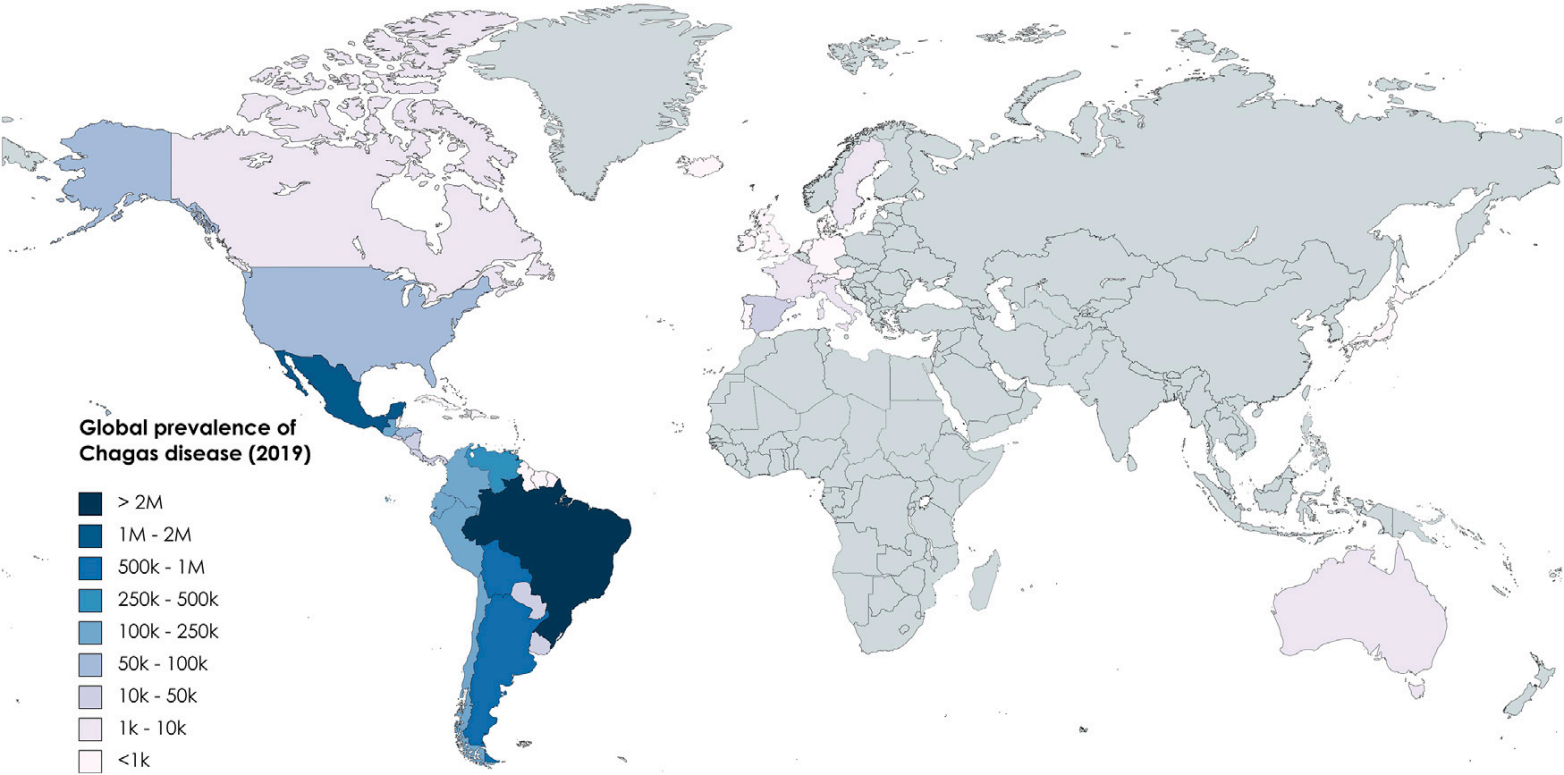
Global prevalence of CD based on data from Global Burden of Disease (2019).

Chagas disease (CD), also known as American trypanosomiasis, is a potentially life-threatening Neglected Tropical Disease (NTD) caused by the protozoan parasite *Trypanosoma cruzi* (*T. cruzi*). An estimated 6 to 7 million people worldwide are infected with *T. cruzi* (WHO, 2023). The disease is found mainly in endemic areas of 21 Latin American countries but with climate change and population movement the impact is spreading to other regions of the globe (Figure 1). CD places a significant direct financial burden on healthcare system and society that has been estimated to exceed $625 million in healthcare costs and 806,170 DALYs (Gómez-Ochoa et al., 2022). However, the full economic cost to endemic communities is much more significant, resulting in a loss of 752,000 workdays annually due to premature deaths, which carries an average financial burden of 1.2 billion dollars per year in the southern countries of Latin America (Ramsey et al., 2014). Additionally, it is currently estimated that <1% of those infected with CD have access to care and treatment (Cucunubá et al., 2017).

**FIGURE 2:**
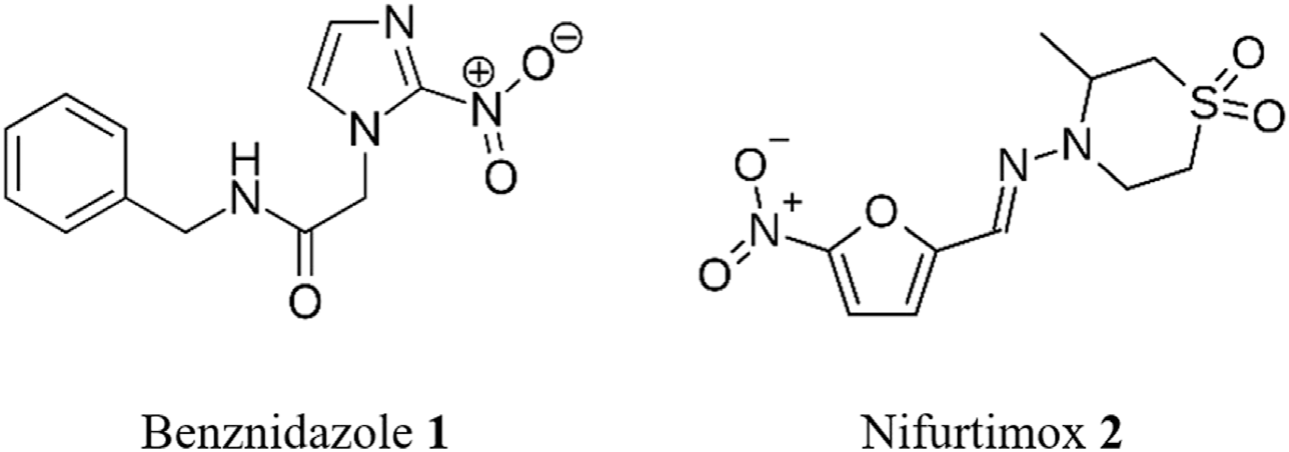
Structure of benznidazole **1** and nifurtimox **2**.

There is no vaccine for CD and there are only two drugs’ treatments approved for this illness. These are benznidazole **1** (BZN), in use since 1971, and nifurtimox **2** (NFX), which was first approved in 1965 (Figure 2). While these old medications remain effective in preventing or curbing disease progression in infected adults, especially those with no symptoms and during the acute infection, they are not without challenges (Crespillo-Andújar et al., 2022). The duration of treatment is long (up to 2 months) and possible adverse reactions can occur in up to 40% of treated adult patients (Jackson et al., 2020), potentially requiring additional treatment for cardiac, digestive, or neurological manifestations. The drugs are contraindicated for certain populations, such as pregnant women and people with kidney or liver failure (Meymandi et al., 2018). Collectively, these factors can contribute to poor patient adherence, which may result in relapse and the development of resistance.

Given this, there is an urgent need for the development of new cost-effective, efficacious drugs. This is challenged by the global resources dedicated to ameliorating the burden of the NTDs. Moreover, even within this limited space, research into CD received only 0.67% of the overall funding allocated to all NTDs over a span of 10 years (2010–2020) (Miranda-Arboleda et al., 2021) reinforcing its reputation as the “most neglected of the neglected tropical diseases” (Zaidel and Sosa Liprandi, 2021).

Drug repurposing (the process of finding new indications for existing drugs) presents a promising approach to addressing this challenge. In this review, we discuss the use of drug repositioning as a cost-effective strategy for the development of new solutions to this devastating disease. We not only explore the latest advances in this approach but also shed light on the challenges that remain for *T. cruzi* chemotherapies.

## 2 Chagas disease

**FIGURE 3:**
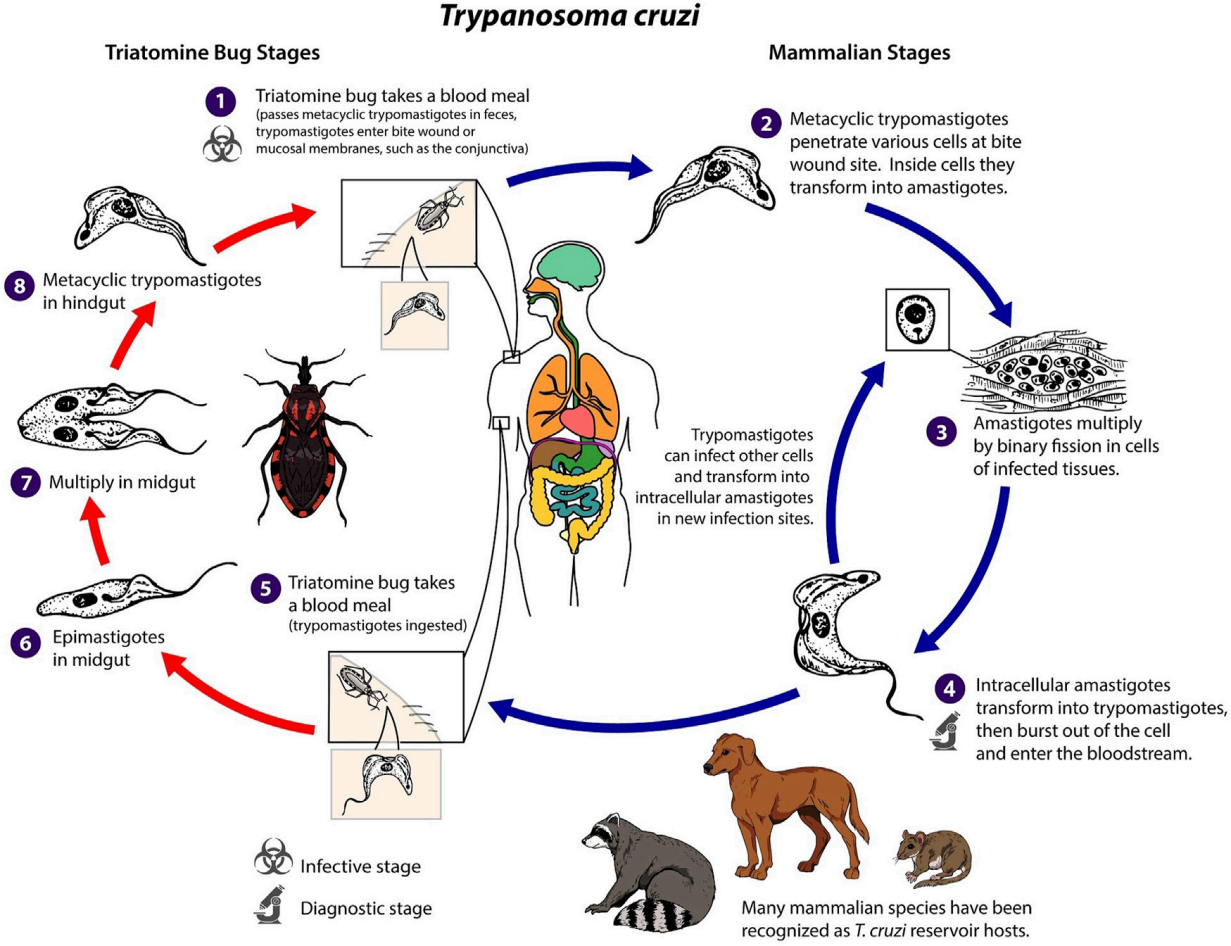
*Trypanosoma cruzi* life cycle adapted from Centers for Diseases Control and Prevention (CDC, 2023).

Identifying and developing new drugs against *T. cruzi* is complicated by the parasite’s complex life cycle (Figure 3). The life cycle has four developmental stages, involving, metacyclic trypomastigotes, epimastigotes, blood-form trypomastigotes, and intracellular amastigotes, which span two host organisms; a triatomine insect, commonly known as a kissing bug, and the mammalian host (Nagajyothi et al., 2012). In humans and other mammals, transmission primarily occurs through insect vectors. This happens when an infected triatomine insect feeds on the blood of a host. Trypomastigotes are released in its faeces near the site of the bite wound. The trypomastigotes can then enter the host’s body through the wound or through intact mucosal membranes, such as the conjunctiva. Alternative modes of transmission can occur through ingestion of contaminated food, mother-to-child transmission and infected blood transfusions and organ transplantation.

Once inside the host, the trypomastigotes invade nearby cells and differentiate into intracellular amastigotes. These amastigotes multiply by binary fission and differentiate back into trypomastigotes, which are then released into the bloodstream. The trypomastigotes can infect cells from various tissues and transform into intracellular amastigotes in new infection sites. Unlike African trypanosomes (*T. brucei*), bloodstream trypomastigotes of *T. cruzi* do not replicate. Replication only resumes when the parasites enter another cell or are ingested by another vector. The cycle is propagated when an uninfected kissing bug feeds on human or animal blood that contains circulating parasites. The ingested trypomastigotes transform into epimastigotes in the vector’s midgut, where they multiply and differentiate. The parasites then differentiate into infective metacyclic trypomastigotes in the hindgut, ready to be transmitted to another host. This life cycle leads to a disease with two distinct phases (PAHO, 2023). Following first infection there is an initial acute phase, which can last for several months, during which a high number of parasites circulate in the blood. This can lead to a range of visible signs, initially a skin lesion or swelling of the eye lids but may involve fever, headache, enlarged lymph glands, muscle pain, breathing difficulties, and abdominal and chest discomfort. However, challenging early diagnosis for the majority of individuals, these symptoms are often mild and not easily attributed to a *T. cruzi* infection. During the chronic phase, the blood population of the parasite falls dramatically, and the parasite reside mainly in the heart and digestive muscles. One to three decades later, up to a third of patients suffer from cardiac disorders (CD is the second leading cause of chronic heart failure in Latin America) and up to 1 in 10 suffer from digestive (typically enlargement of the esophagus or colon), neurological or mixed alterations. Then, the infection can lead to progressive damage to the nervous system and heart muscle, resulting in cardiac arrhythmias, heart failure, and sudden death. This unusually slow progression, often without noticeable symptoms has led CD to be labelled as a “silent and silenced disease” and challenges diagnosis and a recognition of the severity of the problem (WHO, 2023).

## 3 Drug discovery vs. drug repurposing

**FIGURE 4:**
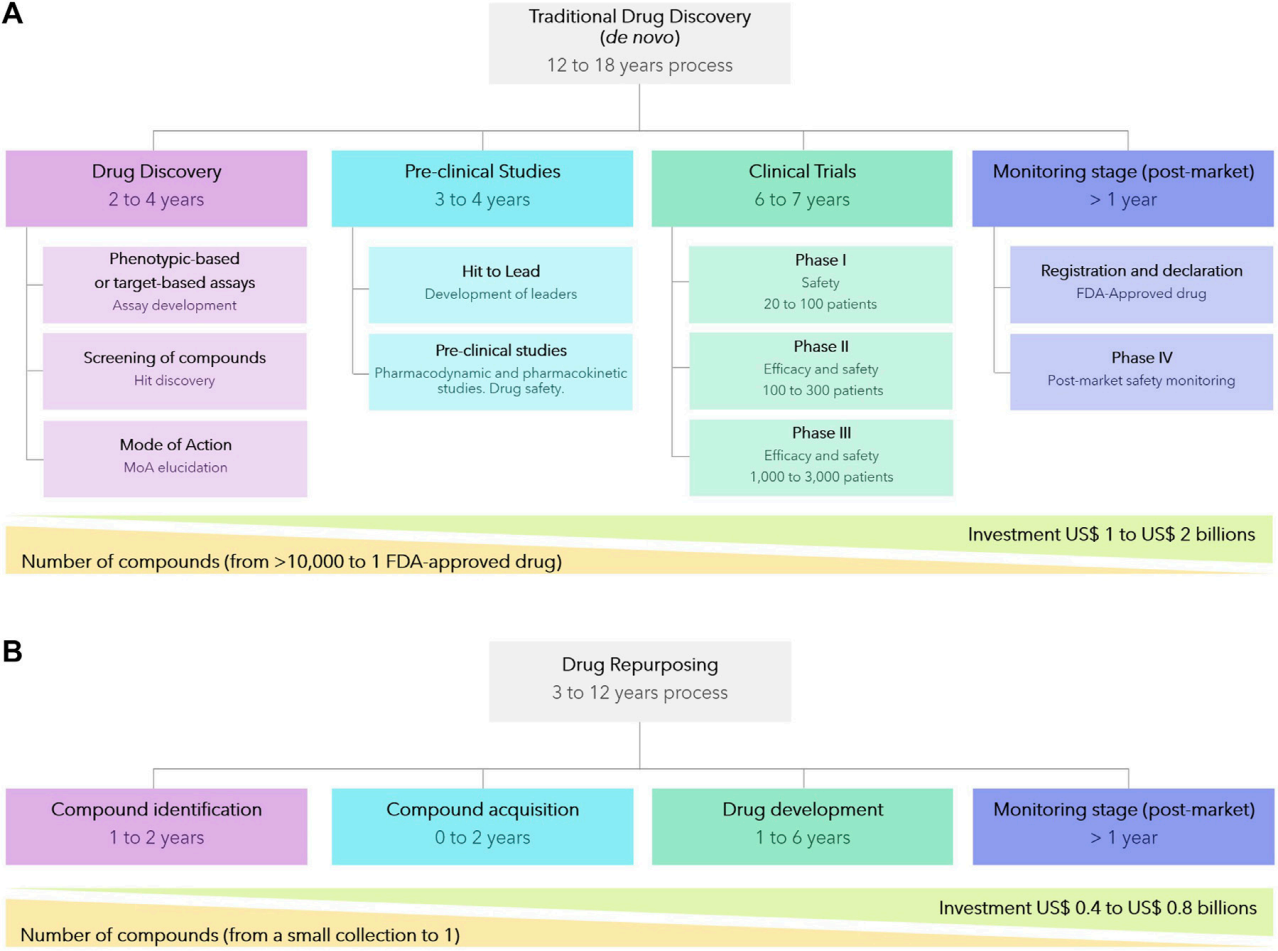
Comparison between *de novo* traditional drug discovery **(A)** and drug repurposing strategies **(B)**.

*De novo* drug discovery, spanning from biochemical concept to the clinic, is an intricate, time-consuming, and expensive journey encompassing several crucial stages. These stages include compound identification, formulation, verification of efficacy and safety in animal models and human volunteers, numerous rounds of pre-clinical and clinical trials, and, ultimately, registration and regulatory approval. Collectively, this is estimated to take between 12–18 years and cost $1–2B to develop a new drug (Figure 4A). Moreover, the probability of failures, most commonly at later more expensive clinical trial stages, are high and this is reflected in the high cost of patented drugs. Consequently, the discovery of new drugs for CD, and other NTDs, for which the average patient exists on an income of less than $2 per day (Clark et al., 2014), is simply not viable for pharmaceutical companies without significant public cross-subsidy.

Drug repurposing, also known as drug repositioning or drug reprofiling, in which compounds developed for one indication are then utilized to provide solutions to an alternative disease, offers various advantages over developing an entirely new drug for a given indication (Pushpakom et al., 2019; Jourdan et al., 2020). Firstly, the risk of failure is lower because the repurposed drug has, if early-stage trials have been completed, already been found to be sufficiently safe in preclinical models and humans. Secondly, the drug development time frame is commonly both shorter and cheaper (Figure 4B) since some preclinical testing, safety assessments, and sometimes even formulation development, will have been completed. Given the reduced costs, drug repurposing is a particularly attractive approach for NTD (Krishnamurthy et al., 2022). Whilst repurposing approaches has been successful applied to afford approved drugs for other diseases, including NTDs such as Human African Trypanosomiasis and Leishmaniasis (where the majority of the current chemotherapy are repurposed drug (Charlton et al., 2018; Braga, 2019), there has yet not been a successful outcome for CD.

## 4 Approaches and techniques for drug repurposing

Strategies for drug repurposing follow traditional models for other drug discovery processes and encompass a combination of molecular and empirical approaches to enhance effectiveness and efficiency. Molecular approaches, commonly known as target-based strategies, primarily rely on hypothesis-driven methods at the single protein level. On the other hand, empirical approaches, known as phenotypic strategies, are centered around evaluating observable measures of response in a whole cell, organism, or system (Schenone et al., 2013; Swinney and Lee, 2020). Both strategies have their advantages and disadvantages that have been discussed in numerous review articles (Swinney, 2013; Moffat et al., 2017).

**FIGURE 5:**
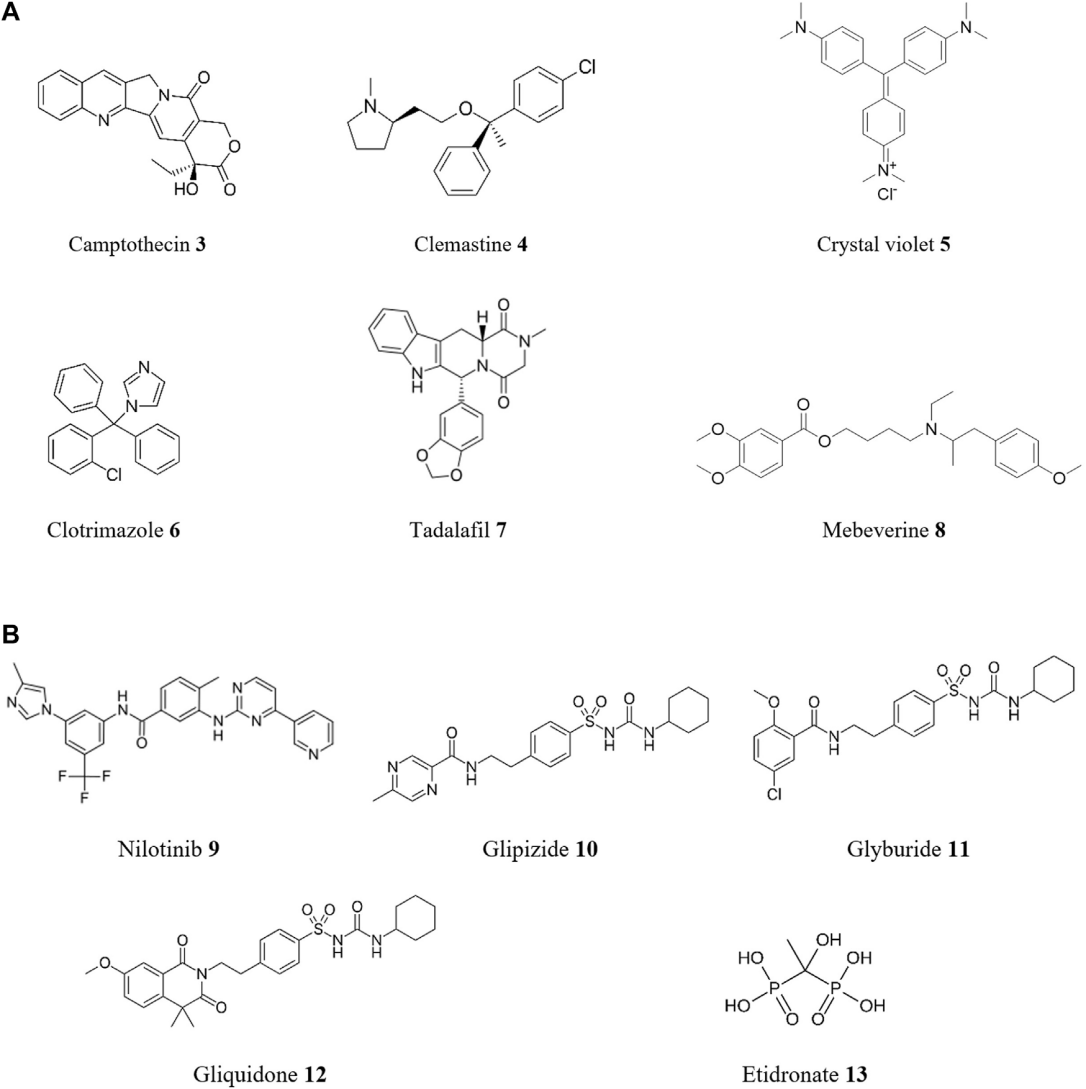
Structures of reported repurposed drugs for CD that have been identified using phenotypic-based approaches [**(A)**, compounds **3** to **8**] or by *in silico* target-based approaches [**(B)**, compounds **9** to **13**].

The process of discovering drugs through phenotypic screening is also referred to as “forward (or classical) pharmacology” or “forward chemical biology”. For instance, a sensitive assay to identify compound activity against *T. cruzi* was developed by Sykes and Avery (2015). This used an image-based assay to estimate the effect of compound treatment on *T. cruzi* amastigotes in 3T3 fibroblasts and host cells. This assay identified active compounds from an in-house FDA-approved drug library and the MMV Malaria Box collection. Prominent compounds include camptothecin **3**, clemastine **4**, crystal violet **5**, and clotrimazole **6** (Figure 5A), all with sub-micromolar activities against this protozoan. In another phenotypic-based assay using a library of 100 registered drugs with drug repositioning potential for NTDs, two compounds with *in vitro* activity against *T. cruzi* were discovered (Kaiser et al., 2015). These compounds were tadalafil **7**, a phosphodiesterase type 5 inhibitor used to treat erectile dysfunction, with an EC_50_ of 8.6 μM and a selectivity index >26 (selectivity index or SI is defined as the ratio of the EC_50_ of host cell versus the EC_50_ of *T. cruzi*), and the antispasmodic mebeverine **8** with an EC_50_ of 3.89 μM and a SI = 18.

In contrast, target-based drug discovery, also known as ‘reverse pharmacology,’ begins with the formulation of a hypothesis that modulation of a specific protein target, believed to be crucial in disease modification, will yield favorable therapeutic effects (Swinney, 2013). The initial step involves the identification and validation of a molecular target, such as an enzyme or receptor, that plays a significant role in the disease process. Subsequently, a range of strategies is employed to discover and develop drugs capable of modulating the target’s activity. These strategies encompass diverse techniques including simple biochemical and biophysical assays, in both single and high-throughput formats (*vide infra*), as well as virtual screening and other *in silico* methods. In the spirit of reducing cost, *in silico* methods which can screen large numbers of drug candidate compounds in a virtual manner using computer generated models of targets or known ligands are ideally suited to repurposing previously reported structures. By employing these techniques, researchers strive to identify innovative therapeutic interventions for CD, aiming to tackle the disease at its molecular roots. Over the past decade, numerous successful cases of drug repurposing for the treatment of CD have been reported using *in silico* approaches (Figure 5B). As an example, a novel virtual screening approach was developed by Juárez-Saldivar et al. (2020) to identify drug repositioning opportunities against *T. cruzi* infection by targeting the bifunctional enzyme dihydrofolate reductase-thymidylate synthase (DHFR-TS). Ten putative *Tc*DHFR-TS inhibitors were identified, including nilotinib **9**, glipizide **10**, glyburide **11**, and gliquidone **12**. These compounds showed growth inhibitory activity against *T. cruzi* epimastigotes. In other study (Valera-Vera et al., 2020), a virtual screening strategy was employed to identify potential inhibitors of enolase, a key enzyme and potential drug target for CD. This *in silico* study proposed etidronate **13**, a bisphosphonate inhibitor of bone resorption, as a potential inhibitor of this molecular target. Due to its safety profile, etidronate **13** provides valuable insights for the development of new drugs targeting *T. cruzi* enolase (*Tc*ENO). However, further exploration of its potential through *in vitro* and *in vivo* studies is necessary.

**FIGURE 6:**
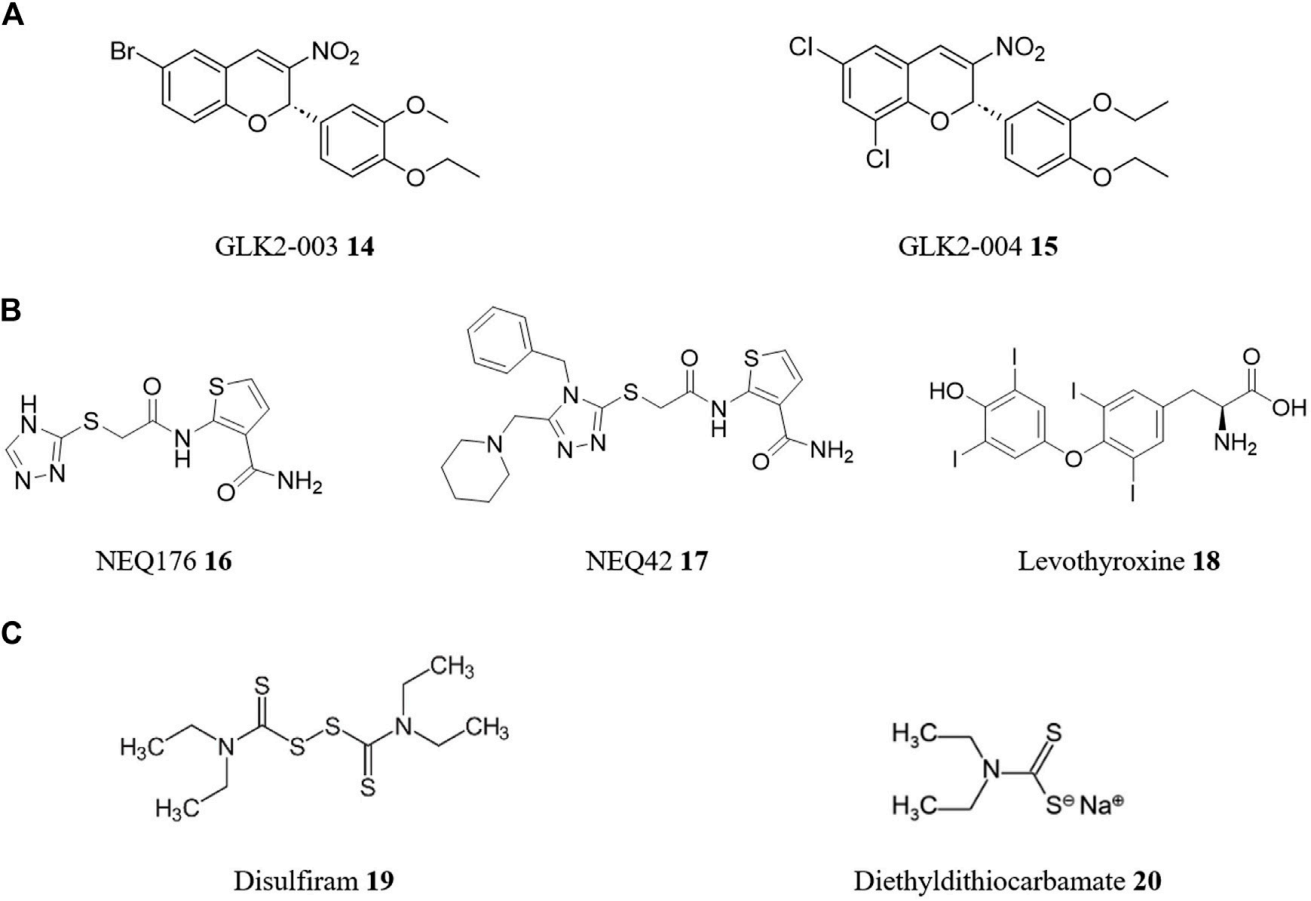
Structures of reported repurposed drugs for CD that have been identified using HTS [**(A)**, compounds **14** and **15**] or by a combination of both target-based and phenotypic-based approaches [**(B)**, compounds **16** to **18**]. Structures of repurposed drugs for combinatory therapy for CD treatment [**(C)**, compounds **19** and **20**].

An important technique in both approaches is high-throughput screening (HTS), which allows researchers to rapidly screen thousands to millions of compounds in a relatively short period of time, greatly accelerating the drug discovery process. In a typical HTS experiment, a library of compounds is screened against a particular molecular target (target-based) or whole cell (phenotypic-based) assay (Blay et al., 2020). For instance, in the search for drug targets against *T. cruzi*, a HTS campaign focused on *Tc*GlcK, a glucokinase enzyme was performed by Mercaldi et al. (2019). Glucokinase and hexokinase are crucial in *T. cruzi*’s metabolic pathways, making their inhibition a promising strategy for drug discovery. Out of 13,040 compounds screened, the HTS campaign found 25 enzyme inhibitors from nine chemical classes. Thirteen compounds displayed low micromolar IC_50_ values for enzyme inhibition, with four showing low toxicity to NIH-3T3 murine host cells and notable *in vitro* trypanocidal activity. These four compounds, belonging to three chemical classes (3-nitro-2-phenyl-2H-chromene, N-phenyl-benzenesulfonamide, and gossypol scaffolds), include two potential hit-to-lead candidates (Figure 6A) from the 3-nitro-2-phenyl-2H-chromene class (namely, GLK2-003 **14** and GLK2-004 **15**), holding promise for further exploration in drug discovery.

However, it is important to note that drug repurposing strategies often incorporate both molecular and empirical approaches in complementary ways. This integrative approach maximizes the chances of success by leveraging the strengths of each strategy. By combining target-based and phenotypic methods, researchers can gain a more comprehensive understanding of the underlying biology, discover new targets or pathways, and optimize drug candidates for efficacy and safety. For instance, cysteine proteases are a class of enzymes that have been validated as drug targets for the development of safe and effective pharmacological agents for CD. One such cysteine protease is cruzain, the major cysteine protease of *T. cruzi*. It has been shown to be essential in various stages of the *T. cruzi* life cycle and is a target of rational drug design for chemotherapy of CD (Ferreira et al., 2022). A study screened the ZINC database for compounds with lead-like properties to find new inhibitors of the cruzain protease (Wiggers et al., 2013). Using consensus scoring, target-based molecular docking, and ligand-based similarity searching, 8,600 lead structures were identified from an initial library of 8.5 million compounds. These were then screened through Glide XP and HQSAR, and the top 5% were visually inspected. 23 compounds were selected for *in vitro* phenotypic-based testing against *T. cruzi*-infected cells, with the top two hits (NEQ176 **16** with an EC_50_ of 108 μM and NEQ42 **17** with an EC_50_ of 10.6 μM—Figure 6B) being structurally characterized by X-ray crystallography to understand their binding modes with cruzain. In a second virtual screening campaign, 163 FDA-approved and investigational drugs identified from the DrugBank database, were screened against cruzipain, another major cysteine protease and validated molecular target of *T. cruzi* (Bellera et al., 2014). In this case, using a combination of target-based and phenotypic-based approaches, levothyroxine **18** (Figure 6B), a drug used in hormone replacement therapy for hypothyroidism, had dose-dependent inhibition of cruzipain (IC_50_ of 38.43 μM) and antiproliferative activity on the *T. cruzi* epimastigotes (EC_50_ of 121 mM).

An intriguing strategy for drug repositioning involves the use of combination drug therapy. Combination therapy or polytherapy is a therapeutic intervention in which more than one medication or modality is used. This approach has become increasingly common in medicine, particularly in the treatment of cancer (Bayat Mokhtari et al., 2017) and infectious diseases (Shyr et al., 2021), as it can offer several benefits over single-drug therapies including enhanced efficacy, commonly greater than simple additive effects (synergistic effect), delaying the development of drug resistance, and reducing the risk of side effects and toxicity. Combining two or more drugs with different mechanisms of action can increase the success rate of drug repositioning by providing an alternative approach to treatment (Sun et al., 2016). For example, disulfiram **19**, a medicine used to treat chronic alcoholism, is currently undergoing Phase I/II clinical trials as combined chemotherapy with BZN **1** for CD (Saraiva et al., 2021). *In vitro* and *in vivo* experiments of BZN **1** with disulfiram **19** and/or its metabolite diethyldithiocarbamate **20** (Figure 6C) demonstrated synergistic effects, inhibiting parasite proliferation in infected macrophages and improved survival rates in infected mice compared to BZN **1** alone (Almeida-Silva et al., 2022). Microscopic analysis revealed structural alterations in the parasites, whilst diethyldithiocarbamate **20** treatment increased reactive oxygen species production.

## 5 Advances on drug repurposing for CD

Given the pressing need for new treatments for CD, significant progress has been made over the past years through drug repositioning efforts. In this review, we present an overview of these works based on the therapeutic origin of the drugs used (for a comprehensive list of the compounds discussed and their biological activities, please refer to Supplementary Table S1; Supplementary Figures S1–S3, in Supplementary Material).

Many natural products exhibit significant therapeutic potential and possess a diverse array of biological properties, including antibacterial, antiprotozoal, antimycobacterial, antileishmanial, antitumor, and anti-human immunodeficiency virus (HIV) activities and represent a valuable and fruitful resource for the discovery and development of potent drugs against a variety of diseases (Álvarez-Bardón et al., 2020; Tempone et al., 2021; Lazarin-Bidóia et al., 2022). As such, there are numerous review studies focusing on the potential use of natural products with effects on *T. cruzi*, providing valuable insights for further investigation (Scotti et al., 2016; Nekoei et al., 2022; Barbosa et al., 2023). For instance, antibiotics such as echinomycin derived from *Streptomyces echinatus*, exhibit notable anti-*T. cruzi* activity at nanomolar concentrations (EC_50_ of 1.1 nM) (Annang et al., 2015). However, reflecting the complexities associated with the use of natural products as repurposing therapies, notably the lack of comprehensive *in vivo* studies and limited progress through clinical phases that hinder their immediate application, they will not be further explored in this review.

In these next sections, we delve into the exciting frontier of FDA-approved drug repositioning towards CD, investigating the rationale behind this approach, the challenges encountered, and the successful examples that have paved the way for the identification of novel therapeutic options. Although the majority of drugs discussed in this review are FDA-approved, we have also included, in certain instances, noteworthy molecules that are currently or have been undergoing clinical assessments, expanding the scope of potential therapeutic options for CD. The ultimate target product profile for CD is a multifactorial issue, but for simplicity in this review is described in terms of enhance efficacy and selectivity, safety and tolerability, and is realized by comparison to BZN **1** (DNDi, 2023). Additionally, we classified the selected examples of drug repurposing for CD in two ways: by approach (target-based or phenotypic-based strategies) and by technique. Within the technique category, we further subcategorize our findings into single drug trials (or small libraries), high-throughput screening, virtual screening, and combination drug therapy.

### 5.1 Repurposing anticancer drugs

Anticancer agents, targeting a wide range of proteins such as kinases, epigenetic regulators, DNA repair enzymes, and proteasomes (Zhong et al., 2021), have emerged as a compelling avenue for repurposing towards CD, attracting considerable attention due to their diverse mechanisms of action and potential for multi-targeted effects. For these reasons, there are many instances of anticancer drugs being repurposed to other diseases, including Alzheimer’s disease (Ancidoni et al., 2021), malaria (Porta et al., 2019), COVID-19 (El Bairi et al., 2020), among others.

**FIGURE 7:**
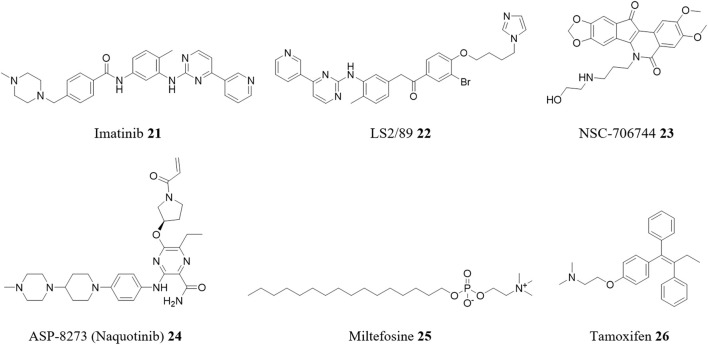
Structures of repurposed anticancer drugs for CD.

The tyrosine kinase inhibitor imatinib **21** (Figure 7), the first small-molecule tyrosine kinase inhibitor (TKI) approved for clinical use by the US Food and Drug Administration (FDA) (Savage and Antman, 2002), was screened for activity against *T. cruzi* (Simões-Silva et al., 2019a). In this phenotypic-based approach, imatinib **21** showed an *in vitro* trypanocidal activity (EC_50_) of 24.8 μM against the intracellular forms of the parasite (SI = 1.5, Y strain) and an EC_50_ of 30.0 μM against the extracellular forms. An additive effect was observed in the combination of imatinib **21** + BZN **1**, in fixed-ratio proportions. Similarly, by using this drug as a pharmacophore in a hit-to-lead strategy, a promising synthetic analogue was developed: LS2/89 **22** (Nesic de Freitas et al., 2023). Although not a repurposed compound, this analogue demonstrated significantly improved potency, with antiparasitic activities more than 100-times lower against the intracellular form (EC_50_ of 0.19 μM) and 10-times lower against the trypomastigote form (EC_50_ of 2.67 μM), compared to imatinib **21**.

In another phenotypic assay, this time in HTS format, screening 7680 compounds from the Repurposing, Focused Rescue, and Accelerated Medchem (ReFRAME) library against *T. cruzi* (CA-I/72) identified seven compounds with potent *in vitro* activity (Bernatchez et al., 2020). These included the DNA topoisomerase inhibitor NSC-706744 **23** with a EC_50_ of 0.44 nM (SI = 214) and the EGFR inhibitor ASP-8273 **24** (naquotinib) with a EC_50_ of 2.7 nM (SI = 191).

Miltefosine **25**, an anti-breast cancer drug already repositioned for leishmaniasis, was evaluated as a monotherapy and in combination with BZN **1** against *T. cruzi* (Gulin et al., 2022). Miltefosine **25** alone showed efficacy against the parasite (VD strain, DTU TcVI) in both *in vitro* (amastigotes and trypomastigotes) and *in vivo* models, with no observed cytotoxic effects on host cells. When combined with BZN **1**, miltefosine **25** exhibited enhanced effectiveness, preventing parasitemia rebound. Given that miltefosine **25** is currently the sole approved oral antileishmanial drug and has demonstrated success in its repositioning for leishmaniasis treatment, it emerges as a highly promising and attractive candidate for further exploration in clinical phases to address CD.

Many drug repositioning studies for CD have shown promising *in vitro* activity profiles. However, there is often a lack of data on subsequent *in vivo* studies, which is crucial as many repositioned drugs fail during this stage. Tamoxifen **26** is an example of this. This antineoplastic drug was tested for its activity against *T. cruzi* and showed promising results *in vitro* against the epimastigote, trypomastigote, and amastigote forms of the CL14, Y, and Y benznidazole-resistant *T. cruzi* strains (Miguel et al., 2010). Tamoxifen **26** demonstrated activity against all life-cycle stages of the parasite, with EC_50_ ranging from 0.7 to 17.9 µM across all strains. However, *in vivo* tests using two experimental models of acute CD showed no significant differences in parasitemia or mortality between tamoxifen-treated and control mice.

### 5.2 Repurposing antihistaminic compounds

**FIGURE 8:**
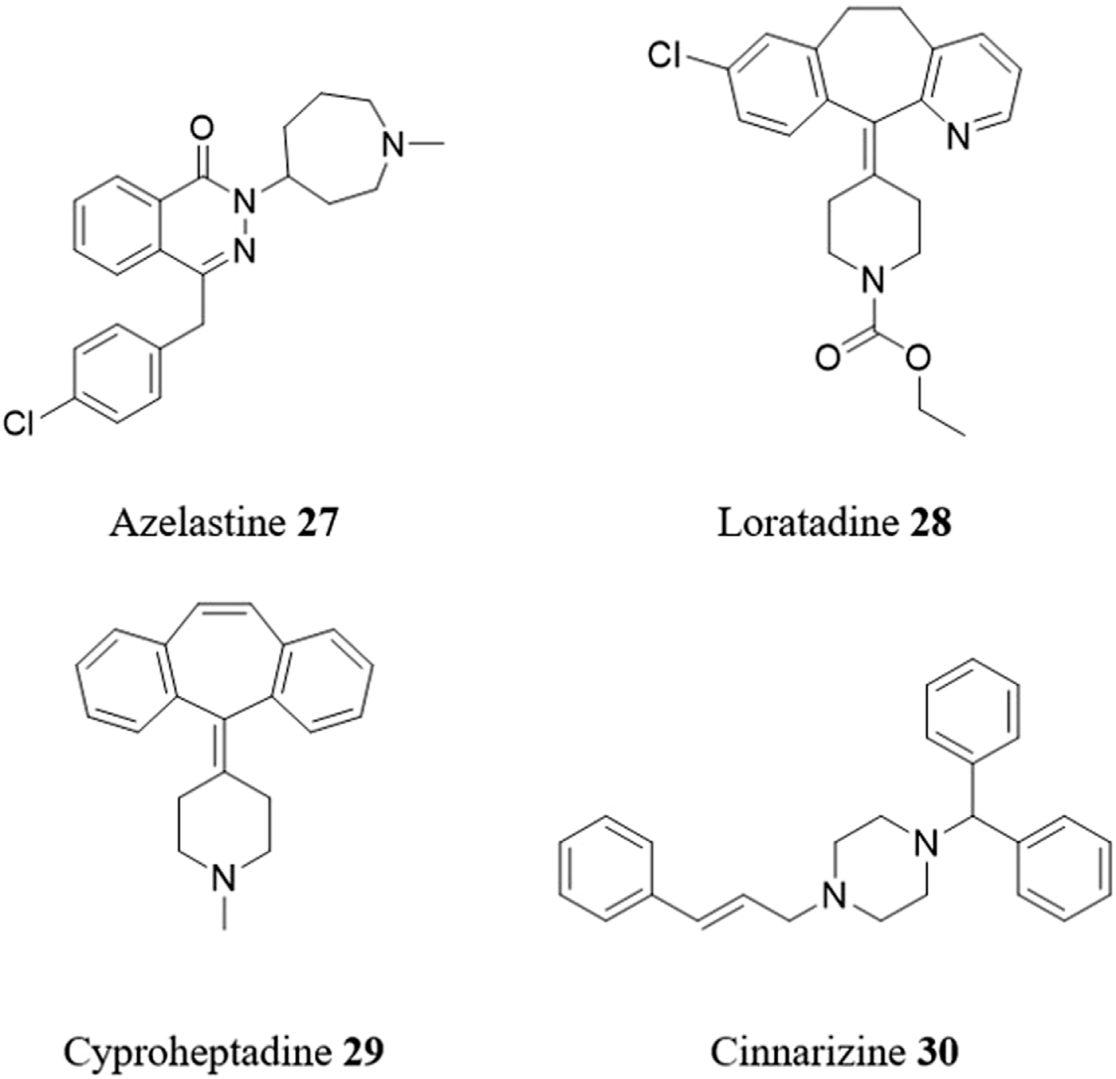
Structures of repurposed antihistaminic drugs for CD.

De Rycker et al. (2016) has described a screening cascade for the identification of compounds with anti-*T. cruzi* activity from 963 clinically tested compounds (from NIH Clinical Collection and the SelleckChem FDA-approved drug library). The cascade includes a primary assay that allows the determination of 14,080 single point measurements (for library screening) or up to 1,280 potency determinations in a single run, and secondary assays to assess static-cidal, rate-of-kill, and cytochrome P450 CYP51 inhibition. A number of promising targets were identified including the first-generation H1 histamine antagonists clemastine **4** and azelastine **27** (Figure 8). Although further optimization is needed due to limitations in their pharmacokinetic and toxicity profiles, these compounds could offer valuable starting points for the development of effective treatments for CD.

The dye crystal violet **5** is known for its ability to eliminate *T. cruzi* in blood banks (Docampo et al., 1983) by inhibiting proline uptake through the proline permease *Tc*AAAP069. However, due to its high cytotoxicity and low selectivity, it is not considered a suitable candidate for drug repositioning. Using ligand based *in silico* drug repurposing, the FDA-approved antihistamines loratadine **28** and cyproheptadine **29** were identified as structurally related compounds to crystal violet **5** (Sayé et al., 2020). These drugs inhibited *Tc*AAAP069 activity and displayed trypanocidal action against all *T. cruzi* life stages in different strains. When combined with BZN **1**, a synergistic effect was observed with loratadine **28** or cyproheptadine **29**.

Alberca et al. (2018) reported a combined ligand- and structure-based virtual screening campaign to find inhibitors of putrescine (a polyamine) uptake in *T. cruzi*. Using an ensemble of linear ligand-based classifiers as an initial screening filter and then docking the results into a homology model of the putrescine permease *Tc*PAT12, the DrugBank and Sweetlead databases were screened for drug repositioning opportunities. As a result, cinnarizine **30**, an antihistamine drug for motion sickness and balance disorder, was selected and tested against *T. cruzi*. Its trypanocidal effects and inhibitory effects on putrescine uptake were confirmed by *in vitro* studies.

### 5.3 Repurposing CNS drugs

**FIGURE 9:**
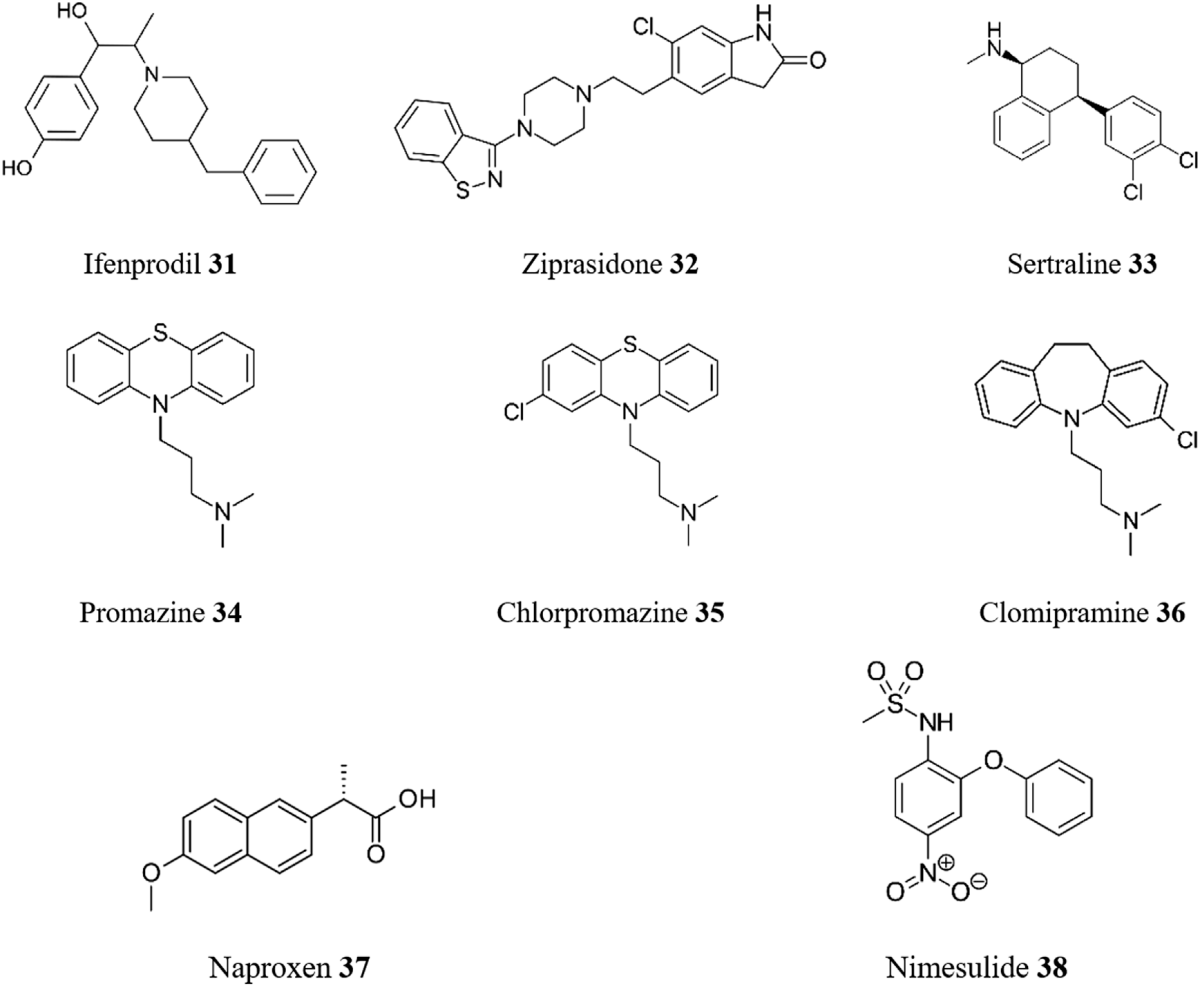
Structures of repurposed CNS drugs for CD.

CNS drugs are medications that affect the central nervous system (CNS). There are many different types of drugs that work on the CNS, including anesthetics, anticonvulsants, antiemetics, antiparkinsonian agents, CNS stimulants, muscle relaxants, narcotic analgesics (pain relievers), nonnarcotic analgesics (such as acetaminophen and NSAIDs), and sedatives. Additionally, pathological studies in CD have confirmed that nodular encephalitis in multiple foci is a key finding in the acute nervous form of the disease (Pittella, 2009). CNS involvement is uncommon in mild cases but can occur in immunosuppressed patients. Ischemic cerebral changes associated with chronic Chagas cardiomyopathy are also common. Given these findings, it is not surprising that the repurposing of CNS medications for the treatment of CD shows promise. For example, ifenprodil **31** (Figure 9), an inhibitor of the NMDA receptor that is currently undergoing clinical trials as a potential repurposed treatment for COVID-19 (Hashimoto, 2021), and ziprasidone **32**, an atypical antipsychotic drug, were identified as promising candidates for drug repurposing through the high content phenotypic screening described above (De Rycker et al., 2016).

In a second example, sertraline **33**, an antidepressant drug, displayed effectiveness against *T. cruzi* strains *in vitro*, with EC_50_ values of 1.4 μM for intracellular amastigotes (Y strain, SI = 17.8) and 14 μM for bloodstream trypomastigotes (Ferreira et al., 2018). Sertraline **33** affected the mitochondrial integrity of *T. cruzi*, decreasing ATP levels. *In silico* approaches using chemogenomic target fishing, homology modeling, and molecular docking suggested *Tc*IDH2 (isocitrate dehydrogenase 2) as a potential target for this drug.

It is well-established that *T. cruzi* relies on the uptake of polyamines from the extracellular medium for survival, therefore, polyamine transporters are promising targets for trypanosomatids (Talevi et al., 2019), as well as other protozoan parasites (Panozzo-Zénere et al., 2018). A ligand-based virtual screening using Ant4, an anthracene-putrescine conjugate inhibitor of the polyamine transport system, as a reference molecule identified three CNS drugs as possible inhibitors of polyamine transport (Reigada et al., 2019a). The antipsychotics promazine **34** and chlorpromazine **35**, and the antidepressant clomipramine **36** were effective inhibitors of putrescine uptake and showed good trypanocidal activity against *T. cruzi* (EC_50_ < 10 μM in all cases). Molecular docking simulations suggest good interactions between the *T. cruzi* polyamine transporter *Tc*PAT12 and these drugs. Additionally, these phenothiazine derivatives have been reported as specific inhibitors of parasite-trypanothione reductase (Iribarne et al., 2009). A study explored combining clomipramine **36** with BZN **1** (García et al., 2016). *In vitro* and *in vivo* tests showed a synergistic effect against *T. cruzi*. During the acute phase, BZN **1** reduced parasitemia, but combining it with clomipramine **36** completely suppressed it. Importantly in the chronic phase, mice treated with both drugs had lower heart damage and inflammation when compared to BZN **1** alone suggesting that this combination shows potential to enable lower BZN **1** doses and concomitant improved safety.

In another case, a structure-based drug repositioning approach was conducted over a set of 20 *T. cruzi* targets to find new treatments for CD (Adasme et al., 2020). The screening yielded over 500 molecules as hits, out of which 38 drugs were prioritized. Compounds showing growth inhibitory activity (<100 μM) when tested on *T. cruzi* trypomastigotes and epimastigotes were selected for further *in vivo* investigation. In mice, a single dose of 100 mg/kg body weight of the nonsteroidal anti-inflammatory naproxen **37** demonstrated the highest inhibition of parasitemia (85.8%) of this collection. For reference, a single dose of at 100 mg/kg body weight of NFX **2** exhibited parasitemia inhibition of 77.8%. Another non-steroidal anti-inflammatory drug nimesulide **38** was identified by Trindade et al. (2021) as a potential candidate for repositioning as a treatment for CD. Treatment of *T. cruzi* epimastigotes with nimesulide **38** resulted in dose-dependent cell death. Nimesulide **38** also inhibited the replication of intracellular amastigotes in *T. cruzi*-infected macrophages. The study suggested that nimesulide **38** affects the parasite’s cell redox balance, leading to cell death primarily through oxidative stress. Ultrastructural changes and a mixed mechanism of cell death involving both apoptosis and necrosis were observed in nimesulide-treated epimastigotes.

### 5.4 Repurposing cholesterol-lowering medicines

*Trypanosoma cruzi* has a high affinity for host lipoproteins and uses the low-density lipoprotein receptor to invade cells. Moreover, Johndrow et al. (2014) has shown that *T. cruzi* infection is associated with an accumulation of low-density lipoprotein and cholesterol in tissues during both the acute and chronic stages of murine CD. This has led to the suggestion that drugs that help lower cholesterol levels in the blood, may have positive effects for CD. The most common anti-cholesterol drugs are the statins, for which the primary target is HMG-CoA reductase. Interestingly, this enzyme is also relevant in the case of *T. cruzi* (Peña-Diaz et al., 2004). However, statins also been shown to possess additional roles including antioxidative, anti-inflammatory, antiatherogenic, and chemotherapeutic activities (Liao and Laufs, 2005; Zhang et al., 2020). Considering these factors together, there have been numerous reports on the repurposing of statins in the context of *T. cruzi*. In this respect, it is noteworthy that the antiparasitic effect of the antifungal ketoconazole (*vide infra*) was improved by lovastatin in a murine model of CD (Urbina et al., 1993). This is proposed to arise through statin mediated reduction of heart inflammation that is commonly observed in chronic *T. cruzi* infection (Guzmán-Rivera et al., 2020).

**FIGURE 10:**
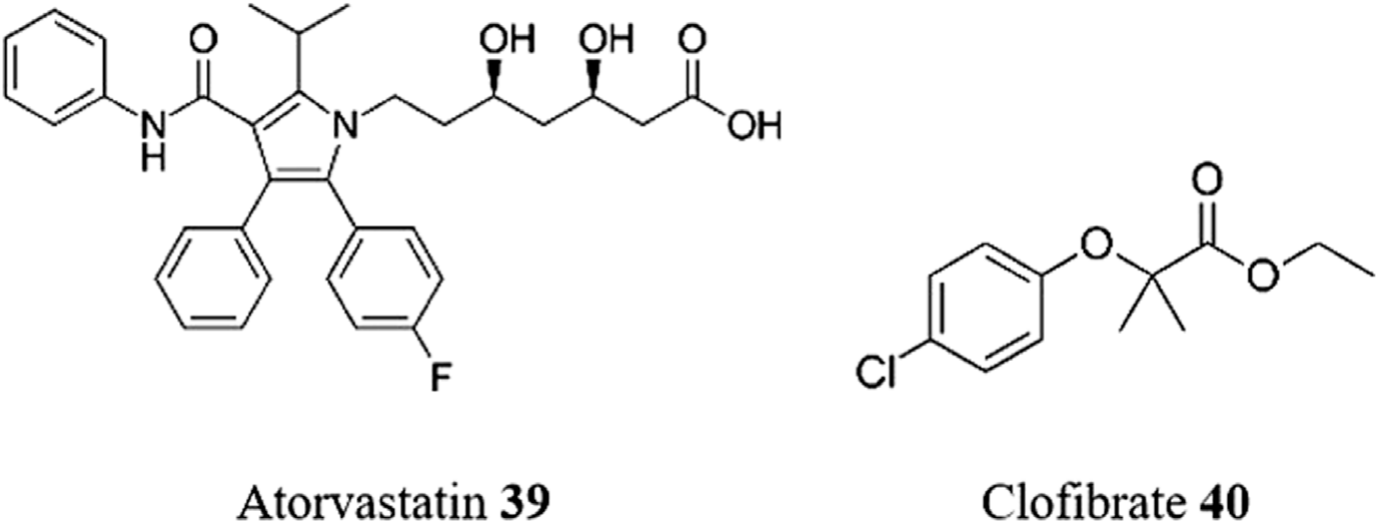
Structures of repurposed cholesterol-lowering medicines for CD.

The activity and selectivity of atorvastatin **39** (Figure 10), an inhibitor of cholesterol synthesis, against *T. cruzi* were evaluated by Araujo-Lima et al. (2018). Atorvastatin **39** showed activity against different strains (Y and Tulahuen) and forms of the parasite (amastigotes and trypomastigotes) with good selectivity in all cases (SI > 20). Combinatory approaches using atorvastatin **39** and BZN **1** in fixed ratio gave synergistic interactions against both trypomastigotes and intracellular forms. Recently, a phase II, multicenter trial to evaluate the impact of atorvastatin **39** on inflammation and cardiac function in patients with chronic CD has been described (Campos-Estrada et al., 2023).

Clofibrate **40**, a lipid-lowering agent commonly used to control high cholesterol and triglyceride levels in the blood, belongs to the class of fibrates. Two groups independently explored its therapeutic potential using an initial virtual screening approach. On one hand, a computer-guided drug repositioning method was employed to identify potential FDA-approved drugs as inhibitors of cruzain, the major cysteine protease of *T. cruzi* (Palos et al., 2017). Through virtual screening of 3180 FDA drugs, clofibrate **40** was selected for *in vitro* and *in vivo* testing. Interestingly, clofibrate **40** emerged as one of the highlighted and selected drugs in the cascade screening conducted by De Rycker et al. (2016), as discussed previously. However, whilst clofibrate **40** exhibited superior activity profiles compared to commercially available drugs (EC_50_ in amastigotes of 6.31 μM), BZN **1** and NFX **2**, in *in vitro* studies, it demonstrated inferior results in short-term *in vivo* studies to reduce parasitemia in infected mice, using a single dose of 100 mg/kg of each drug (reduction to 50% of parasitemia at 6 h for BZN **1**, and a range of 60%–90% for clofibrate **40**).

### 5.5 Repurposing medicines for heart conditions

**FIGURE 11:**
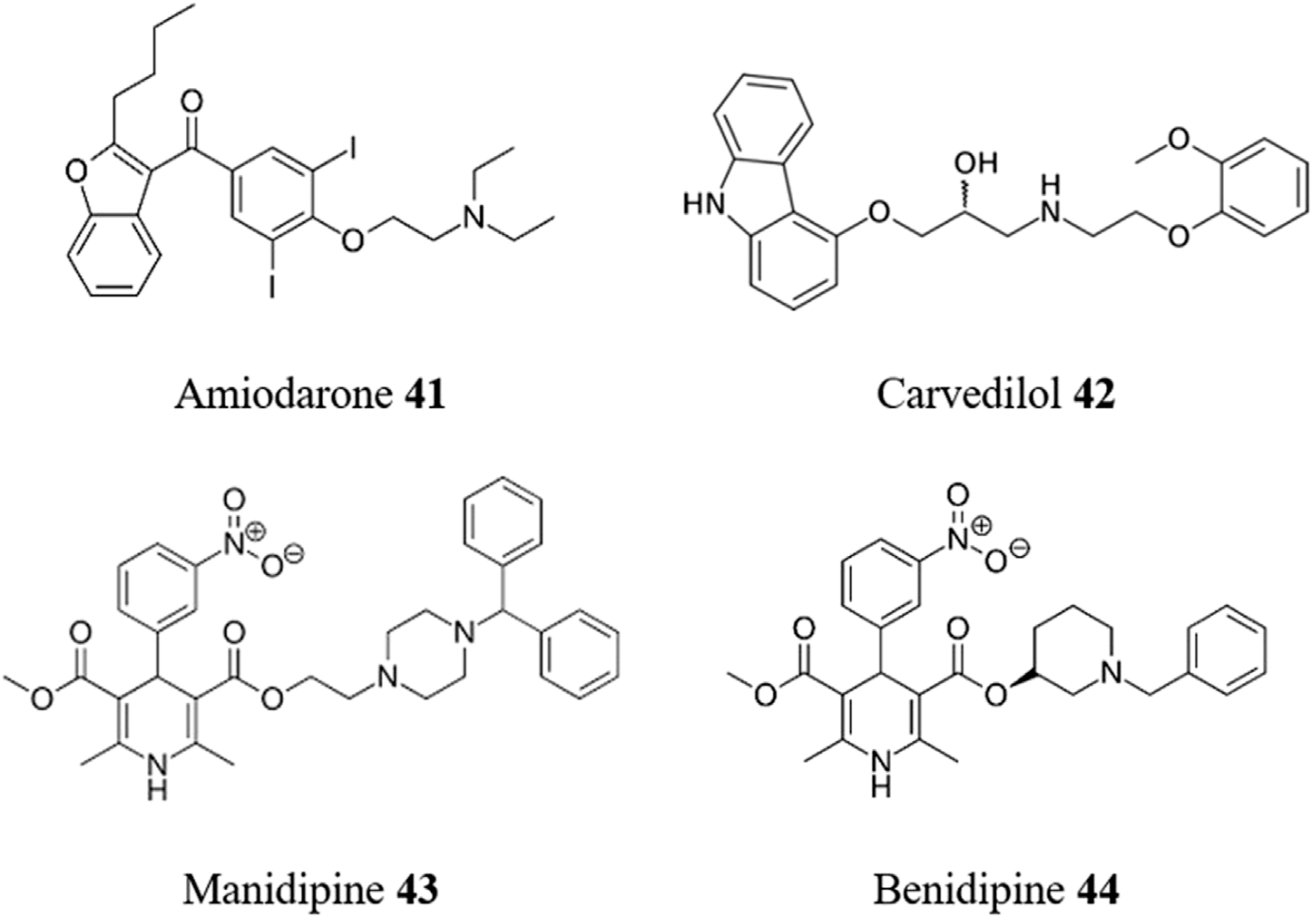
Structures of repurposed medicines for heart conditions toward CD.

Given that CD frequently manifest itself in cardiac failure it is not surprising that repurposing drugs targeted toward other heart conditions has been a popular avenue to explore. The combination of BZN **1** with amiodarone **41** (Figure 11), an antiarrhythmic drug used to treat chronic cardiac CD and also previously recognized as a trypanocidal agent (Adesse et al., 2011), has been investigated (Barbosa et al., 2022). The combined treatment did not improve the direct trypanocidal effect of amiodarone **41** but attenuated the infection-induced cytoskeleton damage of host cells and cytotoxic effects of amiodarone **41**. Therefore, this combination treatment may favor parasite control and limit tissue damage. An alternative coupling of amiodarone **41** with the antifungal itraconazole **51** (*vide infra*) provides an effective treatment of *T. cruzi*-infected dogs and supports the potential therapeutic application of this combination against trypanosomatid infections in humans (Benaim et al., 2021).

Carvedilol **42**, a beta-blocker, selected through virtual screening on the cysteine protease cruzipain of the SWEETLEAD library of approved drugs, promotes the accumulation of immature autophagosomes with decreased acidity and hydrolytic properties by inhibiting the autophagy flux (Rivero et al., 2021). As a result, the viability of trypomastigotes is compromised, and the replication of epimastigotes and amastigotes at 10 μM is hindered, leading to a significant reduction in infection and parasite load. Significantly this effect is maintained in *in vivo* studies with carvedilol diminishing the peak whole-body parasite burden in infected mice.

The antihypertensive drug manidipine **43** was evaluated *in vitro* against *T. cruzi* (Correa et al., 2021) showing potent antiparasitic activity against multiple life cycle stages (EC_50_ of 0.1 μM in amastigotes and 3 μM in trypomastigotes), with promising selectivity against intracellular amastigotes versus the host cell (SI > 1459). Fluorometric analysis showed that manidipine **43** caused depolarization of the plasma membrane and decreased ATP levels, suggesting mitochondrial bioenergetic alteration of the parasite as a potential mode of action.

Finally, benidipine **44**, a calcium channel blocker, has emerged as another promising candidate for repurposing as a trypanocidal drug (Bellera et al., 2015). Discovered by computer-aided screening as a cruzipain inhibitor, this compound underwent comprehensive evaluation, including biochemical and cellular studies, as well as biopharmaceutical, toxicological, physiopathological, and preclinical experiments utilizing an acute model of infection. Remarkably, benidipine **44** demonstrated potent efficacy in reducing parasitemia in an experimental preclinical acute murine infection model at significantly lower doses compared to the positive control, BZN **1** (10 mg/kg/day versus 100 mg/kg/day, respectively). The same research group later found that chronically infected mice treated with this compound showed a reduction in both quantitative and qualitative measures of inflammation compared to untreated mice (Sbaraglini et al., 2016). This was particularly significant in cardiac and skeletal muscle. The reduction in tissue damage can be attributed to the parasiticidal properties of cruzipain inhibitors.

### 5.6 Repurposing antifungal drugs

**FIGURE 12:**
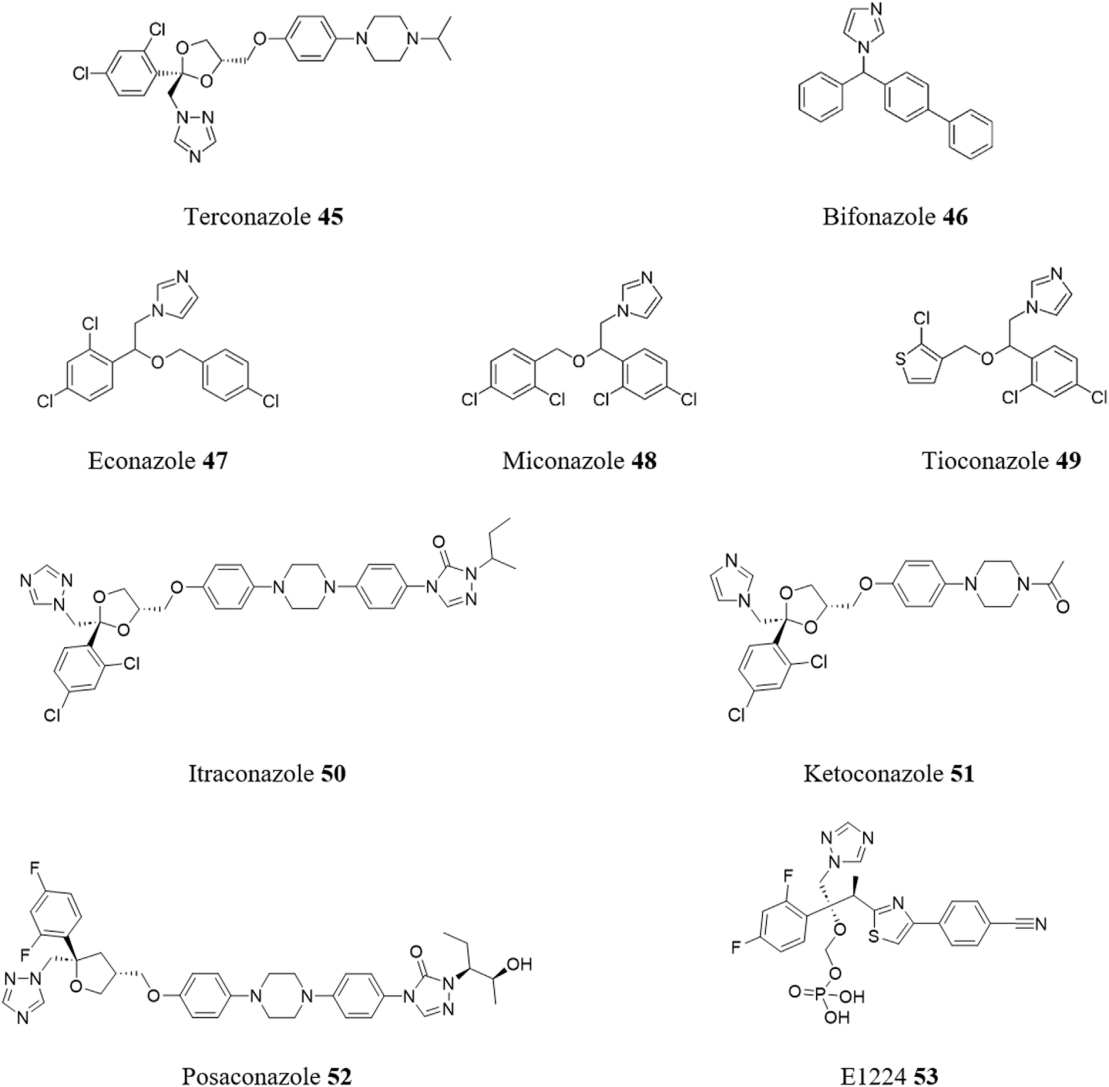
Structures of repurposed antifungal drugs for CD.

Unlike mammalian cells, trypanosomatids primarily produce ergosterol rather than cholesterol, which is similar to the sterol metabolism found in fungi. As such, the sterol biosynthesis pathway presents promising targets for the development of new drugs to treat kinetoplastid infections (Porta et al., 2014; Porta et al., 2023). These targets are sensitive to azoles (Leaver, 2018). These well-known antifungal drugs are already recognized for their activity against *T. cruzi* and act, generally, via inhibition of 14-alpha-sterol demethylase (Buckner and Urbina, 2012). For instance, using a phenotypic-based repurposing strategy, the trypanocidal activity of terconazole **45** (Figure 12), a triazole antifungal drug, was evaluated (Reigada et al., 2019b), revealing trypanocidal activity *in vitro* against epimastigotes of different parasite strains and clinically relevant life-stages of *T. cruzi* (EC_50_ between 4 and 25 μM). Consistent with the above premise, molecular docking simulations suggested that terconazole inhibits *T. cruzi* cytochrome P450 14-alpha-demethylase.

In another case, a collection of 100 registered drugs with repositioning potential for NTDs was gathered and assessed *in vitro* (Kaiser et al., 2015). From a dose-response phenotypic-based screen seven azoles and triazoles were identified as the most potent class of inhibitors from this set of compounds. These active compounds displayed EC_50_ values in the range of 0.003–0.3 μM and SI > 100, and include the imidazoles bifonazole **46**, clotrimazole **6**, econazole nitrate **47**, miconazole **48** and tioconazole **49**, and the triazoles itraconazole **50** and ketoconazole **51**.

However, the failure of two triazole antifungals, posaconazole **52** and E1224 **53**, to demonstrate sustained clearance of *T. cruzi* parasitemia in chronically infected patients in phase II clinical trials (Molina et al., 2014) has challenged the sole use of azoles as a therapeutic class for the treatment of CD. Given this, coupled with the limited efficacy of treatment for CD in the chronic phase, a study was conducted in dogs infected with a benznidazole-resistant strain of *T. cruzi* to test the effectiveness of combining BZN **1** with the antifungal azole itraconazole **50** (Cunha et al., 2022). The dogs were divided into four groups and treated with BZN **1**, itraconazole **50**, a combination of BZN **1** and itraconazole **50**, or left untreated. Over a period of 24 months, the BZN **1** and BZN **1** + itraconazole **50** groups showed negative results in PCR and hemoculture tests, while BZN **1** alone showed partial success. Reactive immunoassay results persisted in all treated animals. Echocardiography and histopathological analysis indicated improved cardiac conditions in the BZN **1** + itraconazole **50** group compared to itraconazole **50** alone. Inflammation and fibrosis were significantly reduced in the BZN **1** + itraconazole **50** group, suggesting an improvement or stabilization of the dogs’ clinical condition.

### 5.7 Repurposing antibacterial and antiviral drugs

**FIGURE 13:**
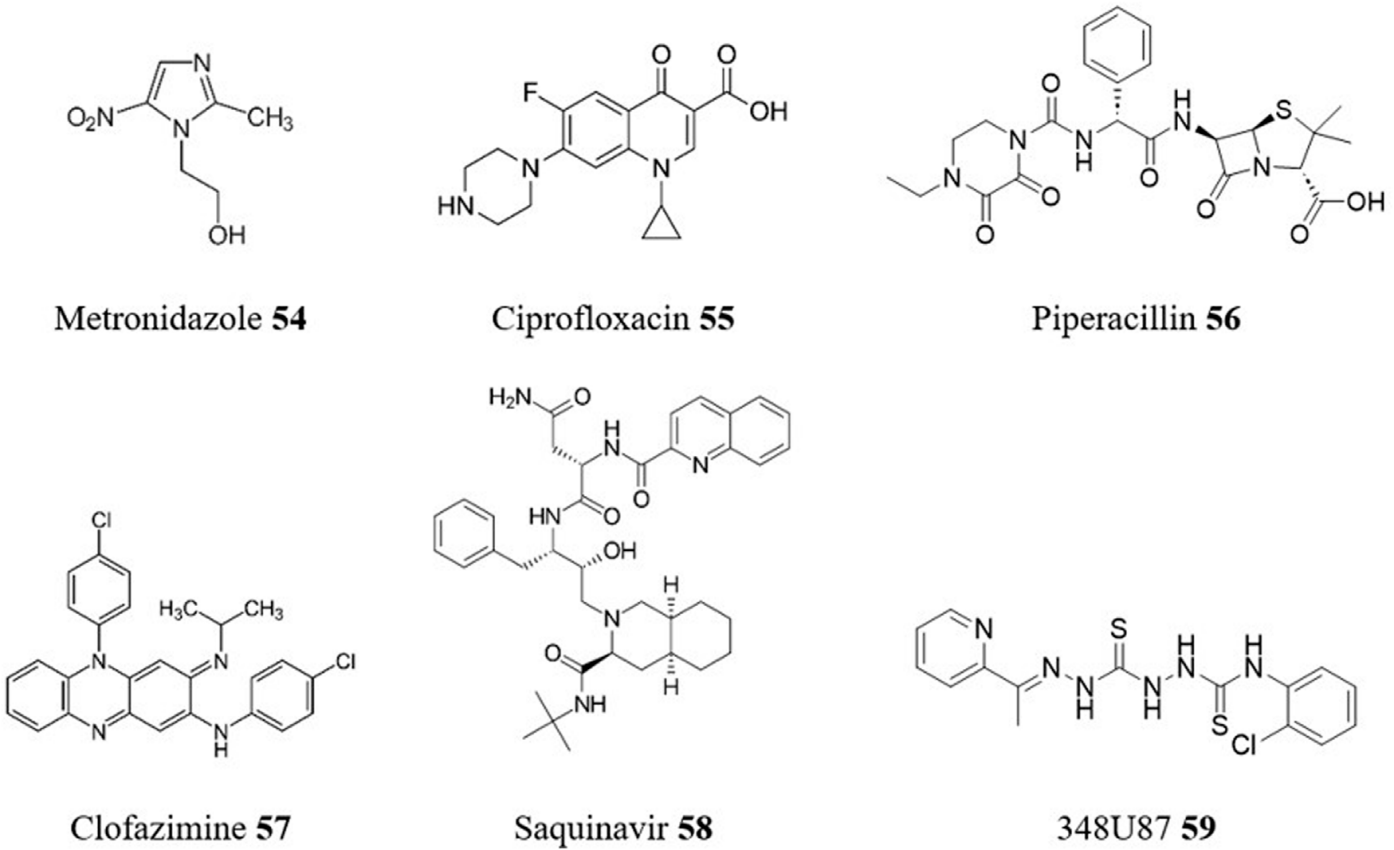
Structures of repurposed antibacterial and antiviral drugs for CD.

Metronidazole **54** (Figure 13) is a broad-spectrum nitroimidazole antibiotic with activity against various parasites including trichomonas and giardia. In *in vitro* repurposing studies, as a monotherapy, metronidazole **54** had low potency against *T. cruzi*, but in combination with BZN **1**, it increased BZN’s efficacy. *In vivo*, metronidazole **54** did not suppress parasitemia but improved survival at certain doses, and the combination therapy with BZN **1** prevented mortality and protected against electric cardiac alterations caused by the parasite (Simões-Silva et al., 2017).

A number of other antibiotics have emerged from various screening campaigns. For example, in the structure-based screening conducted by Adasme et al. (2020), the most effective *in vitro* trypanocidal compound was the fluoroquinolone antibiotic ciprofloxacin **55**. Further evaluation in an *in vivo* experiment, showed that ciprofloxacin **55** demonstrated good levels of inhibition of parasitemia in mice (66.7% in a single dose of ciprofloxacin **55** at 100 mg/kg body weight). Similarly, piperacillin **56**, a β-lactam antibiotic, emerged as the lead candidate from a virtual screening of 3180 FDA-approved drugs conducted by Palos et al. (2017) for potential inhibition of the *T. cruzi* cysteine protease cruzain. Piperacillin **56** exhibited superior *in vitro* trypanocidal activity profiles and comparable results in reducing parasitemia in infected mice during in short-term *in vivo* studies, when compared to the standard drug BZN **1**. A similar target-based virtual screening study identified the antibiotic clofazimine **57**, which finds current use for the treatment of leprosy, as a promising lead structure for the inhibition of proline uptake through the proline permease TcAAAP069 (Sayé et al., 2020).

A number of antiviral drugs have been evaluated for their potential against CD, with mixed results. Saquinavir **58**, an antiretroviral drug, showed potential as a trypanocidal compound in *in vitro* assays (Bellera et al., 2015). However, poor solubility prevented it from advancing to *in vivo* studies in mice. In contrast, the antiherpetic compound 348U87 **59** has demonstrated considerable promise against *T. cruzi* CA-I/72 strain showing a sub-nanomolar EC_50_ (0.63 nM) and a SI of 1294 (Bernatchez et al., 2020).

### 5.8 Repurposing antiparasitic drugs

**FIGURE 14:**
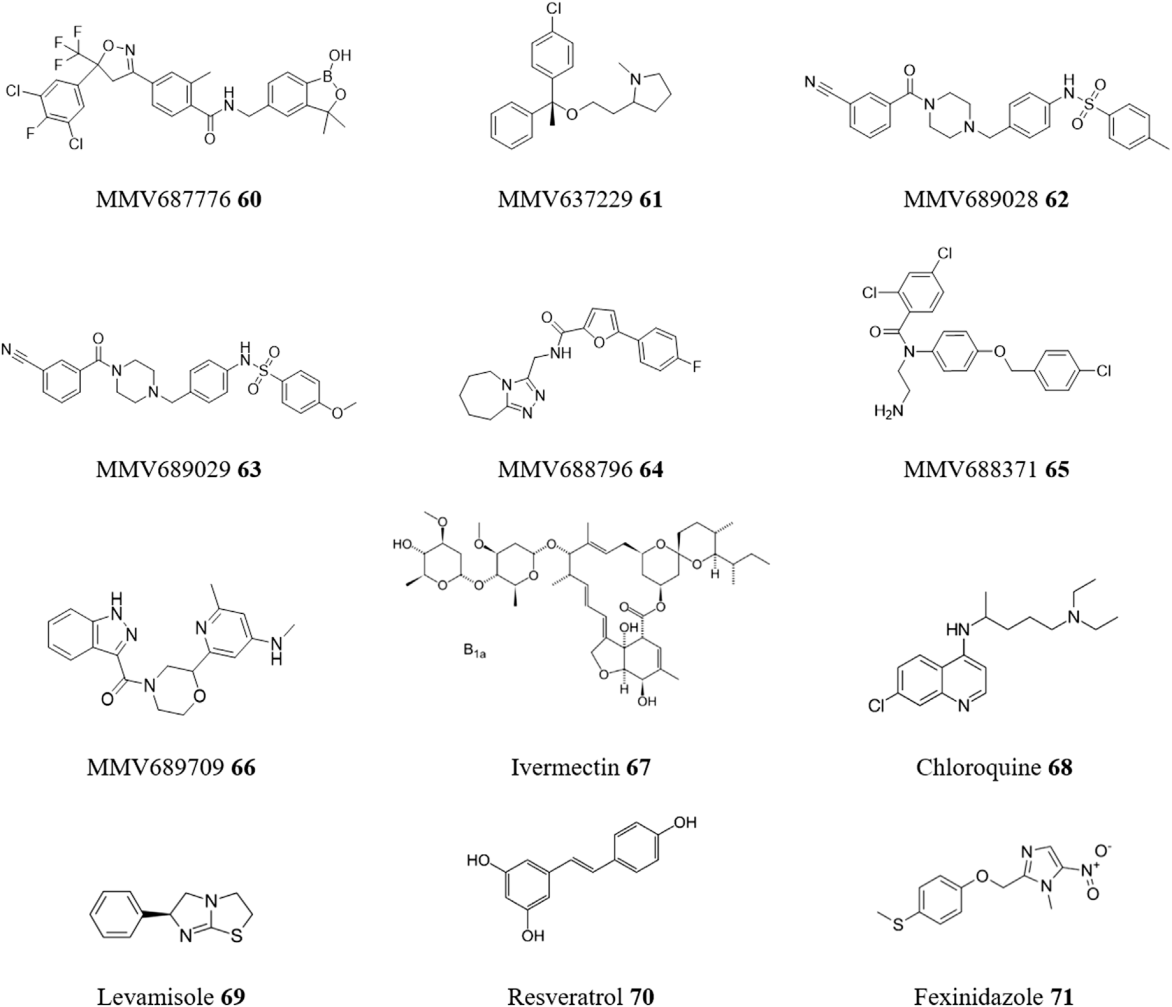
Structures of repurposed antiparasitic drugs for CD.

It is only logical that existing antiparasitic agents are candidates for drug repositioning for treatments for CD. One resource is the open-access Pathogen Box collection provided by the Medicines for Malaria Venture (MMV), (MMV, 2023). Pathogen Box is a collection of 400 compounds, including 200 drug-like and 200 probe-like compounds. These were selected from over 20,000 antimalarial hits from corporate and academic libraries and represent a structurally diverse set. Testing the complete Pathogen Box identified many molecules with good activity (micromolar and sub-micromolar) and SI (>5) in *T. cruzi* phenotypic-based assays (Duffy et al., 2017). Among them, 7 compounds stood out (Figure 14): MMV687776 **60**, MMV637229 **61**, MMV689028 **62**, MMV689029 **63**, MMV688796 **64**, MMV688371 **65**, and MMV689709 **66**. Whilst not formally repurposing, all these structures represent strong starting points for the search for new chemical entities against CD.

In a true repurposing study, the broad-spectrum antiparasitic ivermectin **67** was investigated as a trypanocidal agent against *T. cruzi* (Fraccaroli et al., 2022). In this case, ivermectin **67** affected the proliferation of *T. cruzi* epimastigotes and amastigotes, and the viability of trypomastigotes in a dose-dependent manner, with a SI of 12 for the amastigote stage. However, drug combinations of ivermectin **67** with BZN **1** or NFX **2** showed mainly additive effects. In contrast, a combination of BZN **1** and the anti-malarial drug chloroquine **68** significantly reduced *T. cruzi* infection *in vitro* and was eight times more effective in reducing *T. cruzi* infection *in vivo* than BZN **1** monotherapy. This could enable higher treatment efficacy while mitigating the adverse effects of high doses of BZN **1** (Pandey et al., 2022).

Co-administration of the anthelminthic drug levamisole **69**, with BZN **1** partially reduced parasitemia and slightly promoted animal survival (Simões-Silva et al., 2019b). As levamisole **69** has immunomodulatory activity, and when tested alone did not decrease parasitemia or mortality rates in a murine infection model, this suggest that these effects are related to Th1-response modulation. Similarly, resveratrol **70**, an activator of type III KDACs, which has been shown to exhibit a range of anti-parasitic properties including anti-*T. cruzi* effects (Campo, 2017), also has a host cell response. For example, a resveratrol **70** dosage partially protected mammal cells from infection without activating apoptosis. However, these were *in vitro* experiments and further *in vivo* studies are needed to determine if this therapy could be used as a pre-exposure prophylactic drug (Rodriguez et al., 2022).

Finally, a notable example of drug repositioning is the translational research program by DNDi (Drugs for Neglected Diseases initiative) that repositioned fexinidazole **71** for treatment of CD. Originally developed in the late 1970s as a broad-spectrum anti-infective agent, fexinidazole **71** is a pro-drug of 5-nitroimidazole activated by NTR-1. It was later selected, among other 700 nitroheterocyclic compounds, for development by the DNDi as a treatment for sleeping sickness (Torreele et al., 2010). It has shown superior efficacy in curing experimental *T. cruzi* infections compared to BZN **1** and NFX **2**. It also has been reported for its oral efficacy in acute and chronic experimental models of benznidazole-susceptible, partially resistant, or resistant *T. cruzi* isolates (Bahia et al., 2012). A Phase II clinical trial evaluating its effectiveness, safety, and tolerability in patients with asymptomatic chronic infections was completed at the end of 2022 (Torrico et al., 2023).

## 6 Perspective, challenges, and future directions

In line with the WHO’s roadmap 2021–2030 for eradicating NTDs, and especially in the context of CD, there is an urgent need to expand the range of chemotherapeutic options (Casulli, 2021). The current therapy for this condition is limited, inefficient, and insufficient, consisting of only two outdated drugs. As such, finding a rapid solution is crucial. As evidenced by successes in treating other diseases (Charlton et al., 2018; Hua et al., 2022), coupled with the encouraging results achieved over the past years, summarized in this article, to develop to develop new and improved therapies for CD, drug repurposing offers hope for making progress in the fight against one of the most neglected NTDs.

**FIGURE 15:**
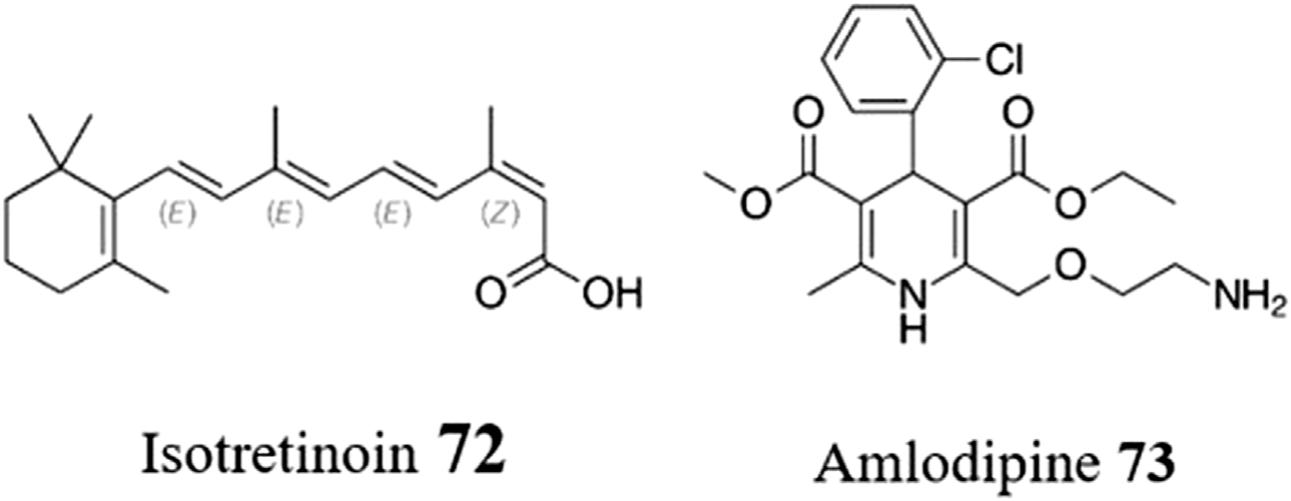
Structure of isotretinoin **72** and amlodipine **73**.

Despite its promise, drug repositioning (and drug discovery in general) in *T. cruzi* faces several challenges, both technical and applied. One major challenge is the lack of comprehensive knowledge of *T. cruzi* biology and drug targets (Jansen et al., 2020). This is exemplified by the extensive morphological and metabolic transformations that occur throughout the various developmental stages of *T. cruzi*, which challenge efforts to develop effective drugs against this parasite (Teixeira et al., 2012). Most notably, the intracellular amastigote can enter in a metabolically quiescent or dormant state which can be non-responsive to otherwise effective trypanocidal drugs (Sánchez-Valdéz et al., 2018; Bhattacharya et al., 2020). As such, there is a pressing need for compounds that can address this chronic state or even restore the sensitivity of the dormant parasite. In this respect, a very recent study by Rial et al. (2023) suggest that isotretinoin **72** (Figure 15), an FDA-approved drug used for severe acne that has previously shown reduction of the trypomastigote burst from infected mammal cells at nanomolar concentrations (Reigada et al., 2017), can also reduce blood parasitemia, prevent negative chronotropic effects and lead to reduced anti-*T. cruzi* antibody levels in murine models of chronic CD.

The success of drug repurposing remains reliant on the availability of existing drugs with well-established safety profiles, which can limit the pool of potential drug candidates. Moreover, this limited pool, and the very nature of repurposing, can mean that innovation and new modes of action are less likely to be revealed. Despite this, there are multitude of libraries available for testing and it is possible to initiate large-scale screening programs that can effectively harness these resources and unlock new treatment options. However, HTS is not cheap and for repurposing it is expected that *in silico* drug-target evaluation will have a growing role. By leveraging computational tools, researchers can predict the activity of existing drugs against specific targets in *T. cruzi*, allowing for the prioritization of compounds for further testing (Trevisan et al., 2020). While this approach is well-established in other diseases, alternative rational methods such as network-based and signature-based approaches have yet to be applied in the context of CD (Bellera et al., 2020). The future application of artificial intelligence (AI) and machine learning to this field holds great promise (Villalta and Rachakonda, 2019).

CD chemotherapy is further challenged by the extensive tissue distribution of *T. cruzi* which requires a similarly wide drug distribution profile in order to clear all parasites (De Rycker et al., 2023). Furthermore, the pharmacological management of this disease is further complicated by the resistance of different parasite strains to currently available drugs, potentially leading to treatment failures (García-Huertas and Cardona-Castro, 2021). One increasingly popular solution to these issues is the use of combination drug therapy, which involves administering multiple drugs with different profiles and modes of action. This can also help address challenges due to drug toxicity. Reflecting this, most combinations that have been reported involve BZN **1** or NFX **2** as the currently approved therapies. However, other repurposing combinations have been explored (Machado et al., 2020; Rocha-Hasler et al., 2021). For instance, combinations of Posaconazole **52** with either amlodipine **73** (Figure 15) or clemastine **4** demonstrated synergistic activity and greater effectiveness in reducing parasitemia levels in mice (Planer et al., 2014). Although attractive for these reasons, developing drug combinations does have hurdles that may not exist for a single repurposed drug, particularly in the assessment of pharmacokinetic and pharmacodynamic parameters as well as efficacy and safety within patients.

In conclusion, repurposing approved drugs presents a fast and appealing approach to address the challenge of CD (Supplementary Table S1, Supplementary Material), with a number of advantages compared to conventional bottom-up drug discovery. Firstly, it significantly reduces the time and cost associated with drug development by leveraging the known safety profiles and pharmacokinetic properties of these drugs, streamlining the regulatory approval process. Secondly, repositioning can rapidly expand the limited treatment options currently available for CD, potentially uncovering additional effective therapies. By exploring diverse mechanisms of action, repurposed drugs may address drug resistance and target different stages of the disease, leading to improved treatment outcomes. Moreover, combining repurposed drugs with existing treatments can result in synergistic effects, enhancing their efficacy in a safer manner. Lastly, utilizing existing drug supply chains facilitates broader access to medications for CD, particularly in resource-constrained regions. Despite these advantages, rigorous research and clinical trials remain essential to confirm the safety, efficacy, and optimal usage of repurposed drugs for CD. Furthermore, the regulatory landscape for drug repurposing is complex, with challenges related to intellectual property, regulatory approval, and commercialization (Halabi, 2018). Despite these minor limitations, whilst not the sole solution, drug repositioning represents a valuable, relatively fast, and cost-effective strategy for developing essential new therapies, particularly for NTDs such as CD.
