# Supplementary material for: Navigating drug repurposing for Chagas disease: advances, challenges, and opportunities
Source: Front Pharmacol. 2023 Jul 27;14:1233253. doi: 10.3389/fphar.2023.1233253 (PMC10416112; doi:10.3389/fphar.2023.1233253)
Supplement: Supplementary file 1 [file DataSheet1.pdf]

Supplementary Material

Navigating Drug Repurposing for Chagas Disease: Advances, Challenges, and Opportunities

Exequiel O. J. Porta,[1,*] Karunakaran Kalesh,[2,3] Patrick G. Steel.[1,*]

[1] Department of Chemistry, Durham University, Durham, United Kingdom.

[2] School of Health and Life Sciences, Teesside University, Middlesbrough, United Kingdom.

[3] National Horizons Centre, Darlington, United Kingdom.

*** Correspondence:** exequiel.o.porta@durham.ac.uk & p.g.steel@durham.ac.uk

Supporting Table 1. Summary of selected reported examples of drug repurposing for CD, including their current uses and biological activities against the *T. cruzi* parasite. References: The Selectivity Index (SI) is defined as the ratio of the toxic concentration of the compound in mammal cells against its effective bioactive concentration in amastigotes of *T. cruzi*. AMA: amastigotes; EPI: epimastigotes; T: trypomastigotes; BT: bloodstream trypomastigotes.

**Drug**	**Reference**	**Original function**	**Biological Activity against *T. cruzi* (EC_{50})**	**Other features**
**1**<br>Benznidazole	Revollo et al., 2019 [1]	Medication used in the treatment of Chagas disease	4.00 μM (AMA)<br>4.02 μM (EPI)<br>5.73 μM (T)	In use since 1971
**2**<br>Nifurtimox	Revollo et al., 2019 [1]	Medication used in the treatment of Chagas disease	2.61 μM (AMA)<br>2.46 μM (EPI)<br>3.60 μM (T)	Approved in 1965
**3**<br>Camptothecin	Sykes and Avery, 2015	Topoisomerase I inhibitor	90 nM (AMA)<br>SI > 56	FDA-Approved drug library

**4**<br>Clemastine	Sykes and Avery, 2015<br><br>De Rycker, 2016	First-generation H1 histamine antagonist	0.29 µM (AMA)<br>SI > 63 (Sykes)<br><br>0.1 µM (AMA)<br>SI = 251 (De Rycker)	NIH and SelleckChem FDA-approved drug library
**5**<br>Crystal violet	Sykes and Avery, 2015<br><br>Sayé et al., 2020	Inhibitor of proline uptake through the proline permease TcAAAP069	90 nM (AMA)<br>SI > 320<br>0.84 µM (T) (Sykes)<br><br>0.3 µM (AMA)<br>0.3 µM (BT)<br>12.7 µM (EPI) (Sayé)	Inhibitor of proline transport (IC_{50} of 7.1µM). Synergistic effect in combination with BZN
**6**<br>Clotrimazole	Sykes and Avery, 2015<br><br>Kaiser et al., 2015	Antifungal	170 nM (AMA)<br>SI > 107 (Sykes)<br><br>6 nM (AMA)<br>SI = 498 (Kaiser)	Inhibitor of the 14-alpha-sterol demethylase
**7**<br>Tadalafil	Kaiser et al., 2015	Phosphodiesterase type 5 inhibitor	8.60 µM (AMA)<br>SI = 26	cGMP-specific 3’-5’-cyclic phosphodiesterase inhibitor
**8**<br>Mebeverine	Kaiser et al., 2015	Antispasmodic	3.89 µM (AMA)<br>SI = 18	Serotonine 5-HT3 receptor agonist
**9**<br>Nilotinib	Juárez-Saldivar et al., 2020	Tyrosine kinase inhibitor (TKI)	6 µM (EPI)	*Tc*DHFR-TS inhibitor
**10**<br>Glipizide	Juárez-Saldivar et al., 2020	Anti-type 2 diabetes mellitus	13.4 µM (EPI)	*Tc*DHFR-TS inhibitor
**11**<br>Glyburide	Juárez-Saldivar et al., 2020	Anti-type 2 diabetes mellitus	66 µM (EPI)	*Tc*DHFR-TS inhibitor
**12**<br>Gliquidone	Juárez-Saldivar et al., 2020	Anti-type 2 diabetes mellitus	12 µM (EPI)	*Tc*DHFR-TS inhibitor
**13**<br>Etidronate	Valera-Vera et al., 2020	Treatment of Paget's disease	No data in this study	Potential *Tc*ENO inhibitor (by docking)

**14** GLK2-003	Mercaldi et al., 2019	TimTec Library	2.1 µM (AMA) SI = 38.4	Inhibitor of *T. cruzi* glucokinase (IC_{50} = 6.1 µM)
**15** GLK2-004	Mercaldi et al., 2019	TimTec Library	2.9 µM (AMA) SI > 36	Inhibitor of *T. cruzi* glucokinase (IC_{50} = 4.8 µM)
**16** NEQ176	Wiggers et al., 2013	Molecule of the ZINC Database	108 µM (T) SI > 2.5 (T)	Cruzain inhibitor with an IC_{50} of 68.5 µM
**17** NEQ42	Wiggers et al., 2013	Molecule of the ZINC Database	10.6 µM (T) SI = 5 (T)	Cruzain inhibitor with an IC_{50} of 21.6 µM
**18** Levothyroxine	Sayé et al., 2020	Synthetic form of the thyroid hormone thyroxine T4	121 µM (EPI)	Cruzipain inhibitor with an IC_{50} of 38.43 ± 6.82 µM
**19** Disulfiram	Almeida-Silva et al., 2022	Drug to treat chronic alcoholism	Pro-drug of diethyl-dithiocarbamate **20**	In clinical trial in combination with BZN
**20** Diethyl-dithiocarbamate	Almeida-Silva et al., 2022	A major metabolite of disulfiram	1.48 µM (EPI)	Synergistic effect combined with BZN (increased the selectivity 13-fold)
**21** Imatinib	Simões-Silva et al., 2019a	Tyrosine kinase inhibitor (TKI)	24.8 µM (AMA) SI = 1.5 30.0 µM (BT)	IMB + BZ in fixed-ratio proportions was additive
**22** LS2/89	Nesic de Freitas et al., 2023	Synthetic analogue of Imatinib	0.19 µM (AMA) SI > 60 2.67 µM (BT)	>10-times better activities than imatinib
**23** NSC-706744	Bernatchez et al., 2020	DNA topoisomerase inhibitors	0.44 nM (AMA) SI = 214	Molecule of ReFRAME library
**24** ASP-8273	Bernatchez et al., 2020	EGFR inhibitor	2.7 nM (AMA) SI = 191	Molecule of ReFRAME library
**25** Miltefosine	Gulin et al., 2022	Anti-leishmanial	0.51 µM (AMA) SI = 112 31.2 µM (BT)	MLT+BZ *in vitro* synergistic effect on trypomastigotes and an additive on amastigote

**26**<br>Tamoxifen	Miguel et al., 2010	Anti-breast cancer	2.7 μM (AMA)<br>0.7 μM (T)<br>12.3 μM (EPI)	No differences in parasitemia and mortality in models of acute CD
**27**<br>Azelastine	De Rycker, 2016	H_1 receptor-blocking (antihistaminic)	0.25 μM (AMA)<br>SI = 63	NIH and SelleckChem FDA-approved drug library
**28**<br>Loratadine	Sayé et al., 2020	Anti-histaminic	13.2 μM (AMA)<br>SI = 5.5<br>12.9 μM (BT)<br>26.4 μM (EPI)	Inhibitor of proline transport (IC_{50} of 23.1μM). Synergistic effect in combination with BZN
**29**<br>Cyproheptadine	Sayé et al., 2020	Anti-histaminic	10.7 μM (AMA)<br>SI = 9.1<br>11.3 μM (BT)<br>52.6 μM (EPI)	Inhibitor of proline transport (IC_{50} of 71.9μM). Synergistic effect in combination with BZN
**30**<br>Cinnarizine	Alberca et al., 2018	Anti-histaminic and calcium channel blocker	6.05 μM (EPI)	Effect on putrescine uptake (52% of initial velocity reduction)
**31**<br>Ifenprodil	De Rycker, 2016	Inhibitor of the NMDA receptor	0.50 μM (AMA)<br>SI = 16	NIH and SelleckChem FDA-approved drug library
**32**<br>Ziprasidone	De Rycker, 2016	Atypical antipsychotic	1.58 μM (AMA)<br>SI = 100	NIH and SelleckChem FDA-approved drug library
**33**<br>Sertraline	Ferreira et al., 2018	Antidepressant of the selective serotonin reuptake inhibitor (SSRI) class	1.4 μM (AMA)<br>SI=17.4 (AMA)<br>14 μM (BT)<br>1.8 μM (T)	Depletion of ATP levels. Affected the parasite TcIDH2 (by in silico).
**34**<br>Promazine	Reigada et al., 2019a	First generation antipsychotic	3.8 μM (AMA)<br>3.4 μM (BT)<br>34.7 μM (EPI)	Inhibitor of *Tc*PAT12 (by docking)
**35**<br>Chlorpromazine	Reigada et al., 2019a	Antipsychotic	1.9 μM (AMA)<br>2.7 μM (BT)<br>41.4 μM (EPI)	Inhibitor of *Tc*PAT12 (by docking)

**36**<br>Clomipramine	Reigada et al., 2019a	Antidepressant	2.9 µM (AMA)<br>1.3 µM (BT)<br>39.7 µM (EPI)	Inhibitor of *Tc*PAT12 (by docking)<br>Synergistic effect combined with BZN
**37**<br>Naproxen	Adasme et al., 2020	Nonsteroidal anti-inflammatory	58.5 µM (BT)<br>>400 µM (EPI)	Inhibition of parasitemia of 85.8% at 8 hours of treatment (*in vivo*)
**38**<br>Nimesulide	Trindade et al., 2021	Non-selective non-steroidal anti-inflammatory drug (NSAID)	12.93 µM (EPI)	Significant changes in cell organelles (ultrastructure). Affected cell redox balance.
**39**<br>Atorvastatin	Araujo-Lima et al., 2018	Statin medication	7.3 µM (AMA)<br>SI = 21 (AMA)<br>7.1 µM (BT)<br>SI = 51 (BT)	Synergistic interactions with BZN against in both trypomastigotes and intracellular forms
**40**<br>Clofibrate	De Rycker, 2016	Lipid-lowering agent	6.31 µM (AMA)<br>SI = 2.5	NIH and SelleckChem FDA-approved drug library
**41**<br>Amiodarone	Barbosa et al., 2022	Antiarrhythmic	0.51 µM (AMA)	BZN+AMD attenuated the infection-triggered cytoskeleton damage of host cells
**42**<br>Carvedilol	Rivero et al., 2021	Beta-blocker	100% growth inhibition at 10 µM (EPI)	Diminishes the peak of whole-body parasite burden in infected mice
**43**<br>Manidipine	Correa et al., 2021	Calcium channel blocker (antihypertensive)	0.1 µM (AMA)<br>SI > 1459<br>3 µM (T)	Decreased ATP levels
**44**<br>Benidipine	Bellera et al., 2015	Calcium channel blocker	19.5 µM (EPI)	Lower parasitemia at doses much smaller than the one used for the positive control BZN
**45**<br>Terconazole	Reigada et al., 2019b	Antifungal	5.9 µM (AMA)<br>4.6 µM (T)<br>25.7 µM (EPI)	Inhibited *T. cruzi* cytochrome P450 14-alpha-demethylase (by docking)
**46**<br>Bifonazole	Kaiser et al., 2015	Antifungal	3 nM (AMA)<br>SI > 1000	Inhibitor of the 14-alpha-sterol demethylase

**47**<br>Econazole	Kaiser et al., 2015	Antifungal	40 nM (AMA)<br>SI = 390	Inhibitor of the 14-alpha-sterol demethylase
**48**<br>Miconazole	Kaiser et al., 2015	Antifungal	4 nM (AMA)<br>SI = 383	Inhibitor of the 14-alpha-sterol demethylase
**49**<br>Tioconazole	Kaiser et al., 2015	Antifungal	64 nM (AMA)<br>SI = 304	Inhibitor of the 14-alpha-sterol demethylase
**50**<br>Itraconazole	Kaiser et al., 2015	Antifungal	4 nM (AMA)<br>SI = 278	Inhibitor of the 14-alpha-sterol demethylase
**51**<br>Ketoconazole	Kaiser et al., 2015	Antifungal	0.27 μM (AMA)<br>SI = 189	Inhibitor of the 14-alpha-sterol demethylase
**52**<br>Posaconazole	Rocha-Hasler et al., 2021	Antifungal	2 nM (AMA)<br>SI > 700	Failed in clinical trials for CD
**53**<br>E1224	Machado et al., 2020	Antifungal	1.84 nM (AMA)<br>SI > 200<br>79.5 nM (T)	Failed in clinical trials for CD
**54**<br>Metronidazole	Simões-Silva et al., 2017	Antibiotic and antiprotozoal	>200 μM (AMA)<br>>200 μM (BT)	Synergistic effect in combination with BZN and prevented mortality (70%)
**55**<br>Ciprofloxacin	Adasme et al., 2020	Fluoroquinolone antibiotic	21.3 μM (BT)<br>>400 μM (EPI)	Inhibition of parasitemia of 66.7% at 8 hours of treatment (*in vivo*)
**56**<br>Piperacillin	Palos et al., 2017	β-lactam antiobiotic	30.6 μM (AMA)	A short-term *in vivo* evaluation showed a reduction of parasitemia in infected mice
**57**<br>Clofazimine	Bellera et al., 2015<br><br>Sayé et al., 2020	Bacteriostatic anti-leprosy	10.6 μM (EPI) (Bellera)<br><br>1.1 μM (AMA)<br>SI = 36<br>2.8 μM (BT)<br>9.3 μM (EPI) (Sayé)	Lower parasitemia at doses much smaller than the one used for the positive control BZN. Inhibitor of proline transport (IC_{50} of 4.3μM). Synergistic effect in combination with BZN.

**58**<br>Saquinavir	Bellera et al., 2015	Protease inhibitors (antiretroviral)	100% growth inhibition at 20 µM (EPI)	Solubility problem
**59**<br>348U87	Bernatchez et al., 2020	Antiherpetic	0.63 nM (AMA)<br>SI = 1294	Molecule of ReFRAME library
**60**<br>MMV687776	Duffy et al., 2017	Anti-lymphatic filariasis	2.39 µM (AMA)	Molecule of the Pathogen Box
**61**<br>MMV637229	Duffy et al., 2017	H1 histamine antagonist	1.22 µM (AMA)	Molecule of the Pathogen Box
**62**<br>MMV689028	Duffy et al., 2017	Originated from the GSK	0.67 µM (AMA)	Molecule of the Pathogen Box
**63**<br>MMV689029	Duffy et al., 2017	Not informed	2.70 µM (AMA)	Molecule of the Pathogen Box
**64**<br>MMV688796	Duffy et al., 2017	Not informed	2.07 µM (AMA)	Molecule of the Pathogen Box
**65**<br>MMV688371	Duffy et al., 2017	Not informed	0.62 µM (AMA)	Molecule of the Pathogen Box
**66**<br>MMV689709	Duffy et al., 2017	Not informed	3.32 µM (AMA)	Molecule of the Pathogen Box
**67**<br>Ivermectin	Fraccaroli et al., 2022	Broad-spectrum antiparasitic	0.3 µM (AMA)<br>SI > 12<br>10.4 µM (T)<br>5.3 µM (EPI)	IVM + BZ in fixed-ratio proportions was additive
**68**<br>Chloroquine	Pandey et al., 2022	Antimalarial	0.4 µM (AMA)	Synergistic effect combined with BZN (2 drugs combination) and with cochicine (3 drugs)
**69**<br>Levamisole	Simões-Silva et al., 2019b	Antihelminthic	No data in this study	Parasitaemia suppression was achieved in combination with BZN
**70**<br>Resveratrol	Rodriguez et al., 2022	Activator of KDACs type III and antioxidant	50.3 µM (T)<br>(Campo, 2017)	Reduced the percentage of infected cells by 50-70% (protective effect)

**71**<br>Fexinidazole	Bahia et al., 2012	Medication used to treat African trypanosomiasis	1.1 μM (AMA)<br>SI > 100	Phase II clinical trial was completed at the end of 2022
**72**<br>Isotretinoin	Reigada et al., 2017	Acne medicine	130 nM (T)<br>SI = 920 (T)<br>30.6 μM (EPI)	Inhibitor of the polyamine transport (TcAAAP). IC_{50} = [4.6–10.3] μM.
**73**<br>Amlodipine	Machado et al., 2020	Calcium channel blocker	2.75 μM (AMA)<br>SI = 33.2 (AMA)<br>4.9 μM (T)	Amlodipine + posaconazole, synergistic effect in mice

References:

[1] Revollo, S., Oury, B., Vela, A., Tibayrenc, M., Sereno, D. (2019). In Vitro Benznidazole and Nifurtimox Susceptibility Profile of Trypanosoma cruzi Strains Belonging to Discrete Typing Units TcI, TcII, and TcV. Pathogens. 8(4):197. doi: 10.3390/pathogens8040197

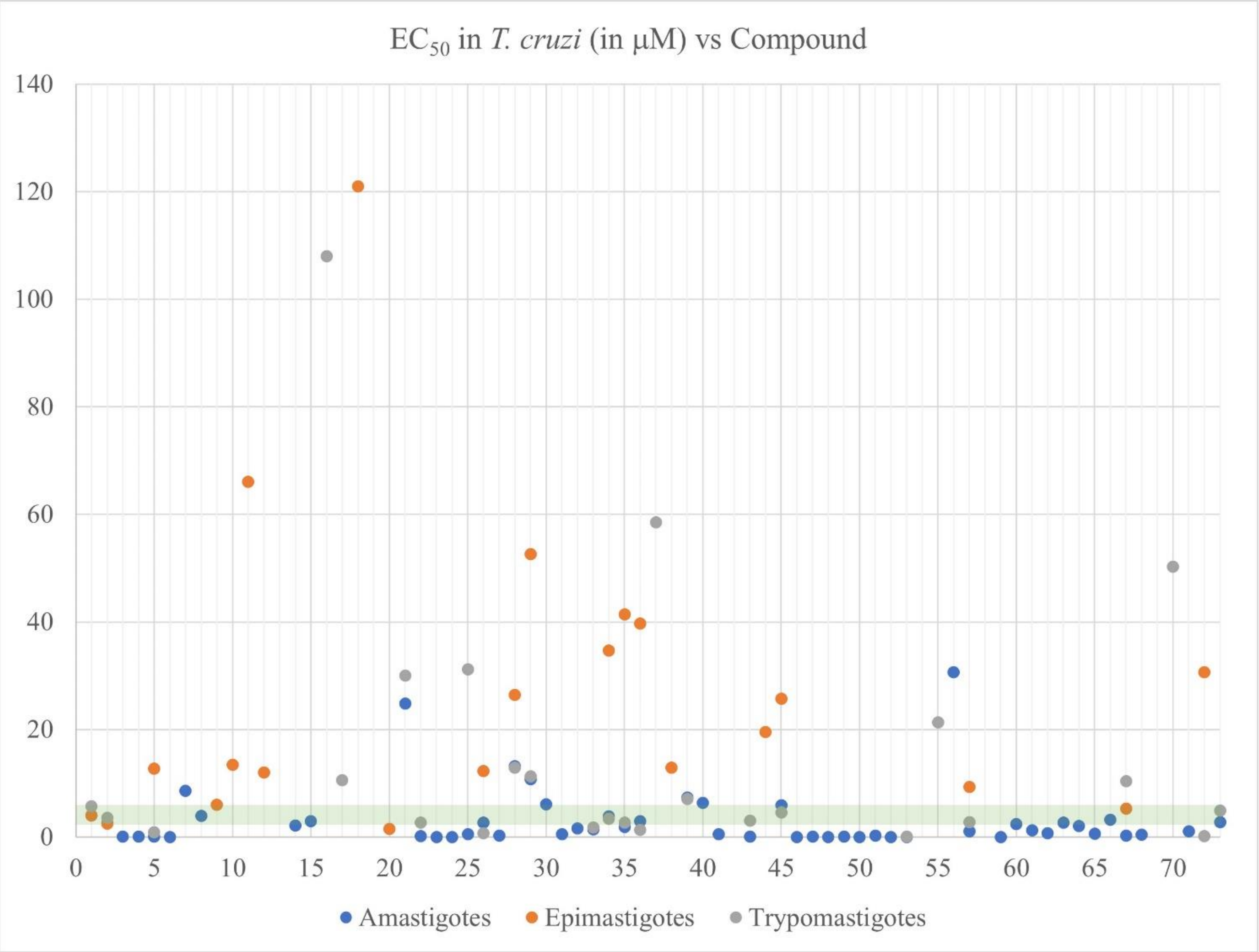

Figure S1. Comparative analysis of the *in vitro* activity of compounds against different stages of *T. cruzi*, quantified by EC_{50} (in µM). Within the green area, lies the range of concentrations where the current chemotherapy agents (Benznidazole **1** and Nifurtimox **2**) are effective *in vitro* against different stages of *T. cruzi*.

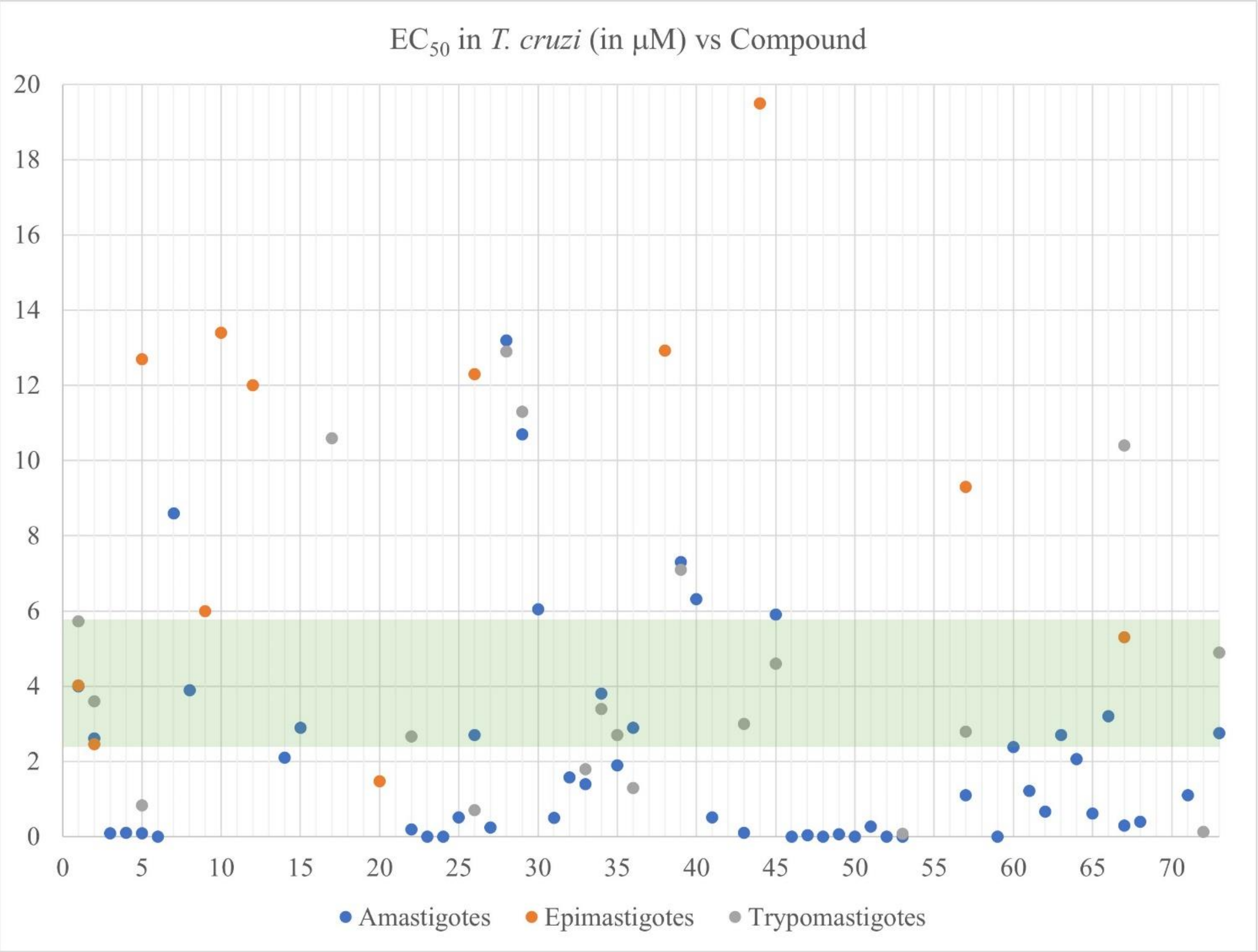

Figure S2. Comparative analysis of the subset of compounds with EC_{50} < 20 µM *in vitro* activities against *T. cruzi* amastigotes, quantified by EC_{50} (in µM). Within the green area, lies the range of concentrations where the current chemotherapy agents (Benznidazole **1** and Nifurtimox **2**) are effective *in vitro* against different stages of *T. cruzi*.

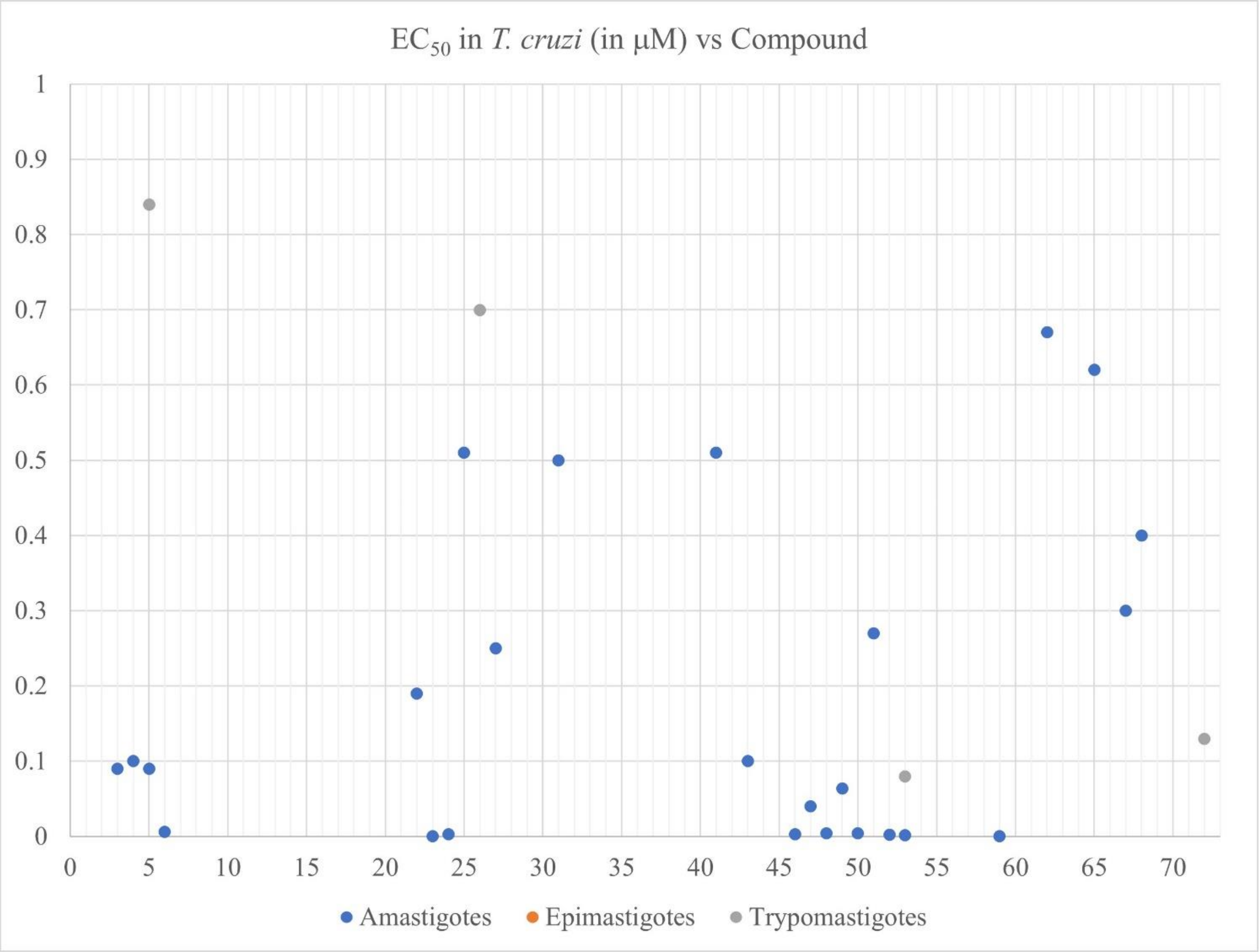

Figure S3. Comparative analysis of the subset of compounds with EC_{50} < 1 μM (submicromolar and nanomolar) *in vitro* activities against *T. cruzi* amastigotes, quantified by EC_{50} (in μM).
